# Supplementary material for: Quantitative Proteomics and Functional Characterization Reveal That Glutathione Peroxidases Act as Important Antioxidant Regulators in Mulberry Response to Drought Stress
Source: Plants (Basel). 2022 Sep 8;11(18):2350. doi: 10.3390/plants11182350 (PMC9500794; doi:10.3390/plants11182350)
Supplement: Supplementary file 1 [file plants-11-02350-s001.zip › Methods S1 and S2.pdf]

Supplemental Methods S1. Assays for antioxidant enzyme activities and GSH content.

Leaf samples (0.5 g) were ground to fine powder in liquid nitrogen, then 5 ml normal saline was added and centrifuged at 12,000 g for 10 min at 4°C. The supernatant was collected and used for assessment of catalase (CAT), guaiacol peroxidase (POD), superoxide dismutase (SOD), ascorbate peroxidase (APX), glutathione S-transferase (GST), glutathione reductase (GR) and GPX activities by using the measure kit (Nanjing Jiancheng Bioengineering Institute, China) for each one of them. The soluble protein content was determined according to the Bradford method [1]. CAT activity was determined by measuring the consumption of H_2O_2 according to the ammonium molybdate method [2]. Superoxide dismutase (SOD) activity was assayed by measuring the ability to inhibit the photochemical reduction of nitro blue tetrazolium (NBT) [3]. One unit of SOD activity was defined as the amount of enzyme per fresh-mass sample causing 50% inhibition of the photochemical reduction of nitro blue tetrazolium. POD activity was measured in accordance with the method of Maehly and Chance (1954) [4]. APX activity was determined following the oxidation of ascorbic acid in absorbance at 290 nm for 1 min according to the method of Nakano and Asada (1981) [5]. The oxidation rate of ascorbic acid was determined from the decrease in absorbance at 290 nm. GR activity was assayed by monitoring glutathione-dependent oxidation of NADPH in absorbance at 340 nm as the method of Smith et al. (1988) [6]. GR activity was expressed as nmol of NADPH oxidized per mg of protein per min. The activity of GST enzymes were measured with CDNB (1-Chloro-2,4-dinitrobenzene) [6]. A unit of activity for both CDNB: GST is defined as the formation of 1nmol product min^{-1} at 25°C. The GPX activity was measured by the amount of GSH consumed per unit time, according to the manufacturer's instructions

(Nanjing Jiancheng Bioengineering Institute, Nanjing, China), in which GSH as the electron donor and hydrogen peroxide as the reaction substrate. All the steps were carried out between 0°C and 4°C. Enzyme activities were assayed using a spectrophotometer (UV-1750, Shimadzu, Japan). We measured the concentration of reduced GSH in leaves using the glutathione assay kit (Nanjing Jiancheng Bioengineering Institute, China). Briefly, leaf tissue and normal saline were added to make homogenate according to the mass volume ratio of 1:9, and supernatant was collected after centrifuged under the condition of ice bath. For GSH content, 0.5 mL of supernatant was combined with 2 mL NaH_2PO_4 (0.1 M, pH=7.4) and 0.5 mL of 5′5′-dithiobis-2-nitrobenzoic acid (DTNB, dissolved in PBS buffer). The absorbance of the solutions was measured at 412 nm and compared to a standard curve to acquire the GSH concentrations. All assays described above were repeated four times, with four biological replicates. The data, which are shown as the means ± SD, were subjected to ANOVA to determine significant differences. The least significant differences (LSDs) of the means were determined via Duncan's test at the level of significance defined as α=0.05.

[1] Bradford MM. A rapid and sensitive method for the quantification of microgram quantities of proteins utilizing the principle-dye binding. Analytical Biochemistry 1976, 72: 248-254.

[2] Peng J, Wang D, Xu C, Chen L, Deng F, Zhang X (2009) Ammonium molybdate method for detecting the activities of rice catalase. Chinese Agricultural Science Bulletin 2009, 25(16): 61-64.

[3] Giannopolitis CN, Ries SK. Superoxide Dismutases: I. Occurrence in higher plants. Plant Physiology 1977, 59(2): 309-314.

[4] Chance B, Maehly AC. Assay of catalases and peroxidases. Methods in Enzymology 1955

2(55): 764-775.

[5] Nakano Y, Asada K. Hydrogen peroxide is scavenged by ascorbate-specific peroxidase in Spinach chloroplasts. Plant and Cell Physiology 1981, 22(5): 867-880.

[6] Anderson JV, Davis DG. Abiotic stress alters transcript profiles and activity of glutathione S-transferase, glutathione peroxidase, and glutathione reductase in Euphorbia esula. Physiologia Plantarum 2004, 120(3): 424-433.

Supplemental Methods S2. Experimental procedures for iTRAQ-based quantitative proteomics.

Protein extraction and trypsin digestion

The samples were ground into cell powder in liquid nitrogen and then transferred to a 5-mL centrifuge tube. After that, four volumes of lysis buffer (8 M urea, 1% Triton-100, 10 mM dithiothreitol, and 1% Protease Inhibitor Cocktail) was added to the cell powder, followed by sonication three times on ice using a high intensity ultrasonic processor (Scientz, CT, USA). The remaining debris was removed by centrifugation at 20,000 g at 4°C for 10 min. Finally, the protein was precipitated with cold 20% trichloroacetic acid (TCA) for 2 h at -20°C. After centrifugation at 12,000 g 4°C for 10 min, the supernatant was discarded. The remaining precipitate was washed with cold acetone for three times. The protein was redissolved in 8 M urea and the protein concentration was determined with BCA kit (Beyotime Biotechnology,

China) according to the manufacturer's instructions.

For digestion, the protein solution was reduced with 5 mM dithiothreitol for 30 min at 56°C and alkylated with 11 mM iodoacetamide for 15 min at room temperature in darkness. The protein sample was then diluted by adding 100 mM TEAB (triethylammonium bicarbonate buffer) to urea concentration less than 2M. Finally, trypsin (Promega) was added at 1:50 trypsin-to-protein mass ratio for the first digestion overnight and 1:100 trypsin-to-protein mass ratio for a second 4h-digestion.

iTRAQ labeling and HPLC fractionation

After trypsin digestion, peptide was desalted by Strata X C18 SPE column (Phenomenex) and vacuum-dried. Peptide was then reconstituted in 0.5 M TEAB and processed according to the manufacturer's protocol for iTRAQ Reagent-8 plex Multiplex Kit (AB SCIEX, Framingham, MA, USA). One unit of iTRAQ reagent were thawed and reconstituted in acetonitrile. The peptide mixtures were then incubated for 2h at room temperature and pooled, desalted and dried by vacuum centrifugation. The tryptic peptides were fractionated into fractions by high pH reverse-phase HPLC using Agilent 300 Extend C18 column (5 μm particles, 4.6 mm ID, 250 mm length). Briefly, peptides were first separated with a gradient of 8% to 32% acetonitrile (pH 9.0) over 60 min into 60 fractions. Then, the peptides were combined into 18 fractions and dried by vacuum centrifuging.

LC-MS/MS analysis

The tryptic peptides were dissolved in 0.1% formic acid (solvent A), and then directly loaded onto a home-made reversed-phase analytical column (15-cm length, 75 μm i.d.). The gradient was comprised of an increase from 6% to 23% solvent B (0.1% formic acid in 98% acetonitrile)

over 26 min, 23% to 35% in 8 min and climbing to 80% in 3 min then holding at 80% for the last 3 min, all at a constant flow rate of 400 nL/min on an EASY-nLC 1000 UPLC system.

The peptides were subjected to NSI source followed by tandem mass spectrometry (MS/MS) in Q ExactiveTM Plus (Thermo Scientific, USA) coupled online to the UPLC. The electrospray voltage applied was 2.0 kV. The m/z scan range was 350 to 1800 for full scan, and intact peptides were detected in the Orbitrap at a resolution of 70,000. Peptides were then selected for MS/MS using NCE setting as 28 and the fragments were detected in the Orbitrap at a resolution of 17,500. A data-dependent procedure that alternated between one MS scan followed by 20 MS/MS scans with 15.0s dynamic exclusion. Automatic gain control (AGC) was set at 5E4. Fixed first mass was set as 100 m/z. The mass spectrometry proteomics data have been deposited to the ProteomeXchange Consortium via the PRIDE partner repository with the dataset identifier PXD010227.

Database search and bioinformatics analysis

The resulting MS/MS data were processed using Maxquant search engine (v.1.5.2.8). Tandem mass spectra were searched against Morus notabilis database concatenated with reverse decoy database. Trypsin/P was specified as cleavage enzyme allowing up to 2 missing cleavages. The mass tolerance for precursor ions was set as 20 ppm in First search and 5 ppm in Main search, and the mass tolerance for fragment ions was set as 0.02 Da. Carbamidomethyl on Cys was specified as fixed modification and oxidation on Met was specified as variable modifications. FDR was adjusted to < 1% and minimum score for peptides was set > 40. The functions and features of quantified proteins were annotated in several different categories, including gene ontology (GO) and KEGG pathway. The quantified proteins were divided into 2 categories. In

this study, a quantitative ratio (treatment/control) over 1.3 was considered up-regulation, whereas a quantitative ratio less than 1/1.3 (0.77) was considered down-regulation ($p < 0.05$). Then, the differentially expressed proteins (DEPs) were classified according to their functions and the significance of the functional enrichment, including protein domain, and KEGG pathway analyzed.
