# Supplementary material for: Quantitative Proteomics and Functional Characterization Reveal That Glutathione Peroxidases Act as Important Antioxidant Regulators in Mulberry Response to Drought Stress
Source: Plants (Basel). 2022 Sep 8;11(18):2350. doi: 10.3390/plants11182350 (PMC9500794; doi:10.3390/plants11182350)
Supplement: Supplementary file 1 [file plants-11-02350-s001.zip › Table S2.pdf]

Table S3. List of 604 differentially expressed proteins (DEPs) in mulberry under drought stress*

Protein ID	Protein description	Gene name	Fold change	P value	Regulation	MW [kDa]	Coverage [%]	Peptides	Unique peptides	PSMs	Subcellular localization
**1. Stress response and defense (59)**											
W9RYR9	late embryogenesis abundant protein 1-like(predicted)	L484_014491	6.58	0.000188	Up	27.9170	9.1	3	3	4	chloroplast
W9S414	Thaumatin(predicted)	L484_021878	6.26	0.000008	Up	35.8830	2.7	1	1	5	nucleus
W9S4V8	low-temperature-induced 65 kDa protein-like isoform X2(predicted)	L484_015784	5.71	0.000055	Up	58.0190	21.2	11	11	34	nucleus
W9RG93	Cold acclimation-induced protein(predicted)	L484_026121	5.48	0.000142	Up	25.9310	4.6	2	2	3	nucleus
W9RGH5	Thaumatin(predicted)	L484_022592	5.21	0.000347	Up	26.0880	39.3	7	4	111	chloroplast
W9R8D6	Thaumatin(predicted)	L484_022590	4.86	0.000038	Up	26.2010	17.2	4	1	51	chloroplast
W9QQI0	Stress response NST1-like protein(predicted)	L484_013493	4.68	0.000129	Up	26.8580	4.7	1	1	1	nucleus
W9QSZ6	Thaumatin(predicted)	L484_024982	4.35	0.000519	Up	27.3600	4.7	1	1	8	chloroplast
W9RUB0	Heat shock cognate 70 kDa protein	L484_025201	4.17	0.000000	Up	71.1980	44.2	30	7	237	cytoplasm
W9RI60	Class I pathogenesis-related protein 4(predicted)	L484_024571	3.24	0.006906	Up	22.1680	15.5	2	1	20	extracellular
W9RMY8	Small heat shock protein HSP(predicted)	L484_003579	3.05	0.000632	Up	10.7520	28.4	2	2	11	cytoplasm
W9RQU8	Pathogenesis-related protein P2	L484_024568	2.81	0.005796	Up	15.7060	9	1	1	3	chloroplast
W9QV79	Heat shock 70 kDa protein	L484_018081	2.28	0.000053	Up	71.7320	36.1	21	5	111	cytoplasm

W9SCV9	Disease resistance RPP13-like protein 4	L484_026733	2.23	0.001672	Up	92.8920	2	1	1	1	cytoplasm
W9QMS5	Heat shock 70 kDa protein	L484_013955	2.14	0.000658	Up	71.6600	28.8	18	1	109	cytoplasm
W9QUF2	Hsc70-interacting protein	L484_018995	1.85	0.000522	Up	41.8340	24.8	8	7	17	nucleus
W9S0L1	Universal stress protein A-like protein	L484_020517	1.81	0.000087	Up	19.6570	24	4	4	7	chloroplast
W9S0W6	Peptidyl-prolyl cis-trans isomerase B	L484_007610	1.78	0.001122	Up	53.4090	5.3	3	3	7	chloroplast
W9QUP4	Peptidyl-prolyl cis-trans isomerase	L484_019172	1.77	0.004932	Up	20.4820	14	2	2	2	cytoskeleton
W9RMT5	Aldehyde dehydrogenase family 2 member	L484_014994	1.71	0.003124	Up	59.3920	31.3	17	12	63	mitochondria
W9QH06	Heat shock protein 83 OS=Morus notabilis	L484_024226	1.66	0.006167	Up	80.5610	13.7	9	1	38	cytoplasm
W9QN32	PREDICTED: late embryogenesis abundant(predicted)	L484_019723	1.58	0.000407	Up	14.6420	39	3	3	12	nucleus
W9SLX9	Universal stress protein A-like protein	L484_007710	1.57	0.007472	Up	17.8540	9.5	2	2	3	chloroplast
W9R4M6	Heat shock protein STI	L484_019004	1.55	0.000580	Up	64.8540	23.9	12	12	27	chloroplast
W9R8X7	Chaperone protein ClpC	L484_005194	0.61	0.003498	Down	62.8790	40.8	21	19	63	chloroplast
W9RJJ7	Chaperone protein ClpC	L484_005193	0.60	0.000961	Down	36.5250	41.2	11	11	33	nucleus
W9SBQ7	Heat shock protein 90	L484_020019	0.59	0.003755	Down	90.0690	7.5	6	3	18	chloroplast,mitochondria
W9SD33	Protein CHAPERONE-LIKE PROTEIN OF POR1-like(predicted)	L484_026719	0.59	0.012857	Down	31.6270	3.9	1	1	1	plasma membrane
W9RXY8	Heat shock cognate protein 80	L484_020213	0.58	0.000133	Down	83.4540	32.5	24	18	79	cytoplasm
W9RVM2	Late embryogenesis abundant	L484_001883	0.57	0.002212	Down	36.0230	3.1	1	1	3	chloroplast

	protein(predicted)										
W9REZ5	Heat shock factor-binding protein 1(predicted)	L484_023534	0.48	0.003073	Down	19.8620	6.6	1	1	2	chloroplast
W9QP55	SHSP domain-containing protein	L484_006034	4.28	0.000144	Up	18.6100	34.1	8	2	29	cytoplasm
W9R4R0	Inactive purple acid phosphatase-like protein(predicted)	L484_024878	2.34	0.004866	Up	50.9450	4.5	2	2	3	nucleus
W9S7V3	Polygalacturonase inhibitor	L484_028064	2.32	0.003973	Up	37.3610	19.8	7	7	14	extracellular
W9S1P9	Hydroxyacylglutathione hydrolase cytoplasmic	L484_017117	2.19	0.000266	Up	28.6140	13.2	2	2	6	cytoplasm
W9RBM4	Light-regulated protein(predicted)	L484_013149	1.97	0.001322	Up	14.8870	34.1	3	3	11	chloroplast
W9RV72	Aquaporin PIP2-7	L484_007653	1.96	0.000398	Up	30.1170	8.5	3	3	4	plasma membrane
W9S6W7	Luminal-binding protein 5	L484_002884	1.92	0.000105	Up	73.1590	8.5	6	2	23	endoplasmic reticulum
W9SID9	Polygalacturonase inhibitor	L484_028063	1.88	0.001234	Up	40.5050	8.9	3	3	5	cytoplasm
W9S2U7	Aquaporin PIP2-1	L484_002027	1.85	0.002249	Up	30.4450	6.6	2	2	9	plasma membrane
W9RX84	Bifunctional protein FolD	L484_006559	1.72	0.000492	Up	40.1030	2.7	1	1	1	chloroplast
W9SBD9	Putative WRKY transcription factor 9	L484_024154	1.67	0.001412	Up	88.3090	3.3	2	2	2	nucleus
W9S667	CBS domain-containing protein CBSX1	L484_027493	1.66	0.002241	Up	25.6710	16.7	4	3	9	chloroplast
W9RM62	Programmed cell death protein 5	L484_010634	1.64	0.009118	Up	21.5350	15.5	3	3	4	mitochondria
W9QN32	PREDICTED: late embryogenesis abundant(predicted)	L484_019723	1.58	0.000407	Up	14.6420	39	3	3	12	nucleus

W9SLX9	Universal stress protein A-like protein	L484_007710	1.57	0.007472	Up	17.8540	9.5	2	2	3	chloroplast
W9QSH7	Aldehyde dehydrogenase family 7 member A1	L484_027409	1.56	0.001766	Up	65.2940	10.9	6	6	16	chloroplast
W9QG08	10 kDa chaperonin	L484_010016	1.55	0.001401	Up	10.6170	56.1	5	5	35	cytoplasm
W9R4M6	Heat shock protein STI	L484_019004	1.55	0.000580	Up	64.8540	23.9	12	12	27	chloroplast
W9RBY6	Bifunctional polymyxin resistance protein ArnA	L484_014097	0.65	0.000693	Down	43.5610	16.5	5	5	10	cytoplasm
W9R8X7	Chaperone protein ClpC	L484_005194	0.61	0.003498	Down	62.8790	40.8	21	19	63	chloroplast
W9RJJ7	Chaperone protein ClpC	L484_005193	0.60	0.000961	Down	36.5250	41.2	11	11	33	nucleus
W9SBQ7	Heat shock protein 90	L484_020019	0.59	0.003755	Down	90.0690	7.5	6	3	18	chloroplast,mitochondria
W9SD33	Protein CHAPERONE-LIKE PROTEIN OF POR1-like(predicted)	L484_026719	0.59	0.012857	Down	31.6270	3.9	1	1	1	plasma membrane
W9RXY8	Heat shock cognate protein 80	L484_020213	0.58	0.000133	Down	83.4540	32.5	24	18	79	cytoplasm
W9RP68	Peptidylprolyl isomerase	L484_015820	0.57	0.002096	Down	16.3250	26.3	3	3	18	chloroplast
W9RVM2	Late embryogenesis abundant protein(predicted)	L484_001883	0.57	0.002212	Down	36.0230	3.1	1	1	3	chloroplast
W9REZ5	Heat shock factor-binding protein 1(predicted)	L484_023534	0.48	0.003073	Down	19.8620	6.6	1	1	2	chloroplast
W9RIV2	Aquaporin TIP1-1	L484_022578	0.21	0.001292	Down	25.8730	4	1	1	3	vacuolar membrane
**2. Cellular redox and antioxidant system (43)**											
W9S7L1	Glutaredoxin domain-containing protein	L484_014582	2.96	0.000474	Up	13.3890	14.4	2	2	2	chloroplast
W9S2N4	Putative	L484_015813	2.85	0.000142	Up	33.7080	11.5	5	1	20	cytoplasm

	quinone-oxidoreductase-like protein										
W9SE23	Peroxidase	L484_018475	2.85	0.000120	Up	36.4880	13.6	4	4	27	chloroplast
W9QHE0	Glutathione peroxidase	L484_024292	2.48	0.000983	Up	18.4420	13.4	2	1	21	cytoplasm
W9QT41	Glutathione peroxidase	L484_025037	2.27	0.003078	Up	18.9620	38.2	8	8	20	mitochondria
W9QH65	Glutathione peroxidase	L484_024293	2.04	0.000421	Up	26.5190	18.6	6	5	45	chloroplast
W9RCX8	Glutathione S-transferase	L484_025426	2.02	0.001778	Up	26.7850	48.3	10	9	74	cytoplasm
W9RT74	Glutathione peroxidase	L484_024694	1.99	0.000374	Up	20.5060	32.1	8	5	21	cytoplasm
W9SDB3	Glutathione peroxidase	L484_025494	1.97	0.000211	Up	26.0130	29.2	7	5	37	chloroplast
W9SDD7	Thioredoxin	L484_026816	1.85	0.000336	Up	13.2220	22.7	3	3	9	chloroplast
W9RLQ1	Putative quinone-oxidoreductase-like protein	L484_015814	1.84	0.006628	Up	35.4180	29.9	10	6	29	cytoplasm
W9QZP8	Glutaredoxin-C5	L484_015915	1.77	0.013193	Up	18.9360	26.1	3	3	9	chloroplast
W9RNB1	Quinone oxidoreductase-like protein	L484_027774	1.70	0.000215	Up	40.6350	56.6	20	17	125	chloroplast
W9S0M4	Quinone oxidoreductase-like protein	L484_020497	1.70	0.000318	Up	34.1020	40.2	13	11	63	chloroplast
W9SBA6	Glutaredoxin domain-containing protein	L484_020532	1.69	0.001711	Up	15.0310	30.8	3	3	34	chloroplast
W9S168	Glutathione S-transferase	L484_027200	1.68	0.000215	Up	39.9490	30.5	12	11	69	chloroplast
W9SW93	Thioredoxin-like 3-1	L484_028031	1.67	0.000097	Up	21.2490	11.5	2	2	3	cytoplasm
W9RMD9	Thioredoxin-like fold containing protein(predicted)	L484_014322	1.66	0.000454	Up	35.4970	13	5	5	17	vacuolar membrane
W9SCK7	Monothiol glutaredoxin-S16	L484_016395	1.57	0.001539	Up	32.5700	14.4	3	3	10	chloroplast
W9QVC2	Peroxiredoxin	L484_007342	1.56	0.004432	Up	22.4790	42.1	7	7	52	chloroplast,mit

											ochondria
W9SEV0	Peroxiredoxin	L484_001936	1.54	0.000664	Up	17.2870	36.4	6	6	44	cytoplasm
W9R4T1	Thioredoxin O1	L484_009848	1.53	0.002092	Up	28.3650	12.3	4	4	13	chloroplast
W9RQI7	L-ascorbate oxidase-like protein	L484_011664	0.64	0.007718	Down	60.3300	4.6	2	2	3	chloroplast
W9SBU2	SodC protein	L484_011779	0.62	0.005244	Down	29.3360	34.2	6	6	89	chloroplast
W9SY79	Hemoglobin-like protein YjbI	L484_019900	1.76	0.003617	Up	19.1320	21.7	3	3	4	nucleus
W9SC39	Peptide methionine sulfoxide reductase A4	L484_017078	1.72	0.000081	Up	29.2280	25.5	6	4	23	chloroplast
W9SL33	Nucleoside diphosphate kinase	L484_001634	1.67	0.000891	Up	16.3330	32.4	5	5	29	cytoplasm
W9SCK7	Monothiol glutaredoxin-S16	L484_016395	1.57	0.001539	Up	32.5700	14.4	3	3	10	chloroplast
W9QSH7	Aldehyde dehydrogenase family 7 member A1	L484_027409	1.56	0.001766	Up	65.2940	10.9	6	6	16	chloroplast
W9QVC2	Peroxiredoxin	L484_007342	1.56	0.004432	Up	22.4790	42.1	7	7	52	chloroplast,mitochondria
W9SEM5	Ferredoxin-thioredoxin reductase, catalytic chain	L484_003491	1.56	0.002258	Up	16.2540	40	6	6	14	chloroplast
W9SEV0	Peroxiredoxin	L484_001936	1.54	0.000664	Up	17.2870	36.4	6	6	44	cytoplasm
W9S327	Peptide-methionine (R)-S-oxide reductase	L484_020754	1.54	0.006269	Up	21.8920	32.8	6	6	9	chloroplast
W9SN39	S-formylglutathione hydrolase	L484_000148	1.54	0.001944	Up	18.5790	34.8	5	5	19	cytoplasm
W9R4T1	Thioredoxin O1	L484_009848	1.53	0.002092	Up	28.3650	12.3	4	4	13	chloroplast
W9SVF9	Monothiol glutaredoxin-S7	L484_015107	0.64	0.006054	Down	20.0390	9.8	1	1	1	chloroplast
W9RQI7	L-ascorbate oxidase-like protein	L484_011664	0.64	0.007718	Down	60.3300	4.6	2	2	3	chloroplast
W9SBU2	SodC protein	L484_011779	0.62	0.005244	Down	29.3360	34.2	6	6	89	chloroplast
W9SCQ6	Ferredoxin--NADP reductase, chloroplastic	L484_026672	0.58	0.000415	Down	39.9310	53.5	19	19	91	chloroplast

W9REH7	2Fe-2S ferredoxin-type domain-containing protein	L484_026246	0.56	0.005824	Down	22.1390	13.6	2	2	5	chloroplast
W9RYH9	PGR5-like protein 1A(predicted)	L484_023150	0.54	0.001608	Down	36.2260	4.3	2	2	2	chloroplast
W9RYX2	Thioredoxin reductase	L484_011273	0.53	0.004546	Down	56.9680	8.1	3	3	4	chloroplast
W9QRL1	Ferredoxin	L484_022337	0.52	0.012335	Down	16.5170	11.1	1	1	8	chloroplast
**3. Photosynthesis (78)**											
W9R536	Chlorophyll a-b binding protein, chloroplastic	L484_002856	0.63	0.017226	Down	28.1100	42.3	7	1	55	chloroplast
W9SG48	Chlorophyll a/b binding protein domain containing protein(predicted)	L484_023403	0.55	0.001639	Down	28.2790	13.8	3	3	3	chloroplast
W9SVH5	Photosystem I reaction center subunit XI	L484_015127	0.53	0.004807	Down	23.1370	10.1	3	3	4	chloroplast
W9SMB1	Chlorophyll a-b binding protein, chloroplastic	L484_017723	0.48	0.001538	Down	29.5390	37.7	8	8	26	chloroplast
W9R7D8	Photosystem I reaction center subunit III	L484_025917	0.44	0.002206	Down	24.7260	19.8	4	4	14	chloroplast
W9R983	Chlorophyll a-b binding protein, chloroplastic	L484_002539	0.43	0.009359	Down	32.8640	3.4	1	1	1	chloroplast
W9R4W5	Chlorophyll a-b binding protein, chloroplastic	L484_002855	0.42	0.000188	Down	28.0730	51.3	7	1	36	chloroplast
W9SDQ4	Photosystem II D2 protein OS=Morus notabilis	L484_000839	0.41	0.002629	Down	34.0530	5.9	1	1	2	plasma membrane
W9R1X7	Chlorophyll a-b binding protein, chloroplastic	L484_006490	0.41	0.013522	Down	27.5560	6.8	1	1	2	chloroplast
W9SID3	Photosystem II 22 kDa protein,	L484_004387	0.40	0.000059	Down	29.6220	15.4	4	4	9	chloroplast

	chloroplastic(predicted)										
W9SBB4	Chlorophyll a-b binding protein, chloroplastic	L484_024124	0.40	0.003051	Down	27.4540	13.2	3	3	9	chloroplast
W9RGN8	Chlorophyll a-b binding protein, chloroplastic	L484_018739	0.39	0.001298	Down	28.5320	13.3	3	3	7	chloroplast
W9RZD3	Chlorophyll a-b binding protein, chloroplastic	L484_013420	0.36	0.002447	Down	26.5770	13.9	4	4	9	chloroplast
W9QYC0	Chlorophyll a-b binding protein, chloroplastic	L484_026409	0.35	0.001186	Down	30.5470	33.8	7	4	17	chloroplast
W9QJH4	Photosystem II CP47 reaction center protein	L484_001781	0.34	0.000226	Down	50.6330	28.7	12	12	67	plasma membrane
W9QWB6	Photosystem II CP43 chlorophyll apoprotein	L484_024765	0.31	0.000280	Down	18.0550	35.2	7	7	39	chloroplast
W9SZU4	Chlorophyll a-b binding protein, chloroplastic	L484_020016	0.27	0.002296	Down	28.4650	18.5	5	2	28	chloroplast
W9S2Y9	Chlorophyll a-b binding protein, chloroplastic	L484_015572	0.27	0.001366	Down	31.4490	19.9	5	2	13	chloroplast
W9SNV8	Photosystem II protein D1	L484_000029	0.24	0.000775	Down	34.7130	11	3	3	26	plasma membrane
W9S9S7	Chlorophyll a-b binding protein, chloroplastic	L484_012656	0.24	0.000903	Down	39.2790	30.6	8	8	36	chloroplast
W9R3W2	Photosystem II D2 protein(predicted)	L484_021838	0.24	0.002639	Down	8.1721	13.9	1	1	4	chloroplast
W9QNL6	Photosystem I P700 chlorophyll a apoprotein A1(predicted)	L484_010617	0.23	0.003068	Down	9.7429	14.1	1	1	4	nucleus
W9QRQ6	Photosystem Q(B) protein	L484_001783	0.14	0.001156	Down	17.4690	5.9	2	2	3	cytoplasm

W9QFY6	Ribulose bisphosphate carboxylase/oxygenase activase 1	L484_016996	3.57	0.000021	Up	52.3840	34.3	17	7	90	chloroplast
W9RSX5	Soluble inorganic pyrophosphatase	L484_008915	1.95	0.000681	Up	30.3880	4.4	1	1	2	chloroplast
W9S8I7	Succinate dehydrogenase [ubiquinone] iron-sulfur subunit, mitochondrial	L484_017682	1.66	0.001262	Up	31.3940	16.1	5	5	11	mitochondria
W9RTY4	Cytochrome c oxidase subunit 5C	L484_019796	1.64	0.040960	Up	6.9651	12.7	1	1	5	cytoplasm
W9RKU9	Germin-like protein	L484_022674	1.61	0.013879	Up	22.9140	6.5	2	2	5	extracellular
W9RUK5	Complex 1 LYR protein(predicted)	L484_015251	1.59	0.000427	Up	10.7530	17.9	2	2	3	chloroplast
W9RQC1	Iron-sulfur cluster assembly protein	L484_015026	1.53	0.000830	Up	17.6710	18.5	3	3	6	mitochondria
W9RD35	Succinate-semialdehyde dehydrogenase	L484_019242	1.53	0.000502	Up	44.2630	5.1	2	2	3	chloroplast
W9RCD5	Succinate-semialdehyde dehydrogenase (Acetylating)	L484_013106	1.52	0.000557	Up	49.2620	6.9	3	3	4	chloroplast
W9SFU1	Mg-protoporphyrin IX chelatase	L484_003164	0.66	0.006775	Down	83.0500	5.1	4	3	6	chloroplast
W9S8P5	Carbonic anhydrase	L484_020509	0.66	0.001381	Down	35.8390	30.7	8	7	60	chloroplast
W9QZ73	Delta-aminolevulinic acid dehydratase	L484_013359	0.65	0.003237	Down	46.7540	9.5	3	3	9	chloroplast
W9RWR9	Uroporphyrinogen-III synthase	L484_026089	0.64	0.019144	Down	24.1870	8.3	2	2	3	chloroplast
W9R816	Dihydrolipoamide acetyltransferase component of pyruvate dehydrogenase complex	L484_016448	0.64	0.001208	Down	58.4810	6.3	3	1	8	chloroplast
W9SDQ0	ATP synthase subunit beta	L484_000834	0.64	0.001767	Down	17.0370	41.8	5	5	48	cytoplasm
W9R0C1	Cytochrome b6-f complex iron-sulfur subunit	L484_022215	0.64	0.000976	Down	24.3760	25.4	5	5	57	chloroplast

W9SL82	Ribulose bisphosphate carboxylase large chain	L484_000836	0.64	0.002135	Down	60.9520	26.3	22	22	413	cytoplasm
W9RGJ1	Transaldolase(predicted)	L484_019027	0.63	0.008022	Down	43.5410	10.9	4	4	5	chloroplast
W9R536	Chlorophyll a-b binding protein, chloroplastic	L484_002856	0.63	0.017226	Down	28.1100	42.3	7	1	55	chloroplast
W9QQ77	Photosynthetic NDH subunit of subcomplex B(predicted)	L484_013517	0.62	0.008606	Down	55.8990	6	2	2	2	chloroplast
W9RNP2	Phosphoenolpyruvate carboxylase 2	L484_010342	0.62	0.006373	Down	111.0500	10.3	9	3	17	cytoplasm
W9SI76	Magnesium-protoporphyrin IX monomethyl ester [oxidative] cyclase	L484_027977	0.60	0.002143	Down	47.3290	8.6	3	3	3	chloroplast
W9QR60	Photosystem I reaction center subunit V	L484_023779	0.59	0.002009	Down	17.4220	21.9	3	3	13	chloroplast
W9RT02	Ribose-phosphate pyrophosphokinase 4	L484_021391	0.59	0.008394	Down	35.6250	3.8	1	1	2	cytoplasm
W9R3K5	Apocytochrome f	L484_003629	0.58	0.001680	Down	28.4050	41.5	10	10	46	mitochondria
W9QNJ2	Protoporphyrinogen oxidase	L484_013889	0.56	0.000707	Down	58.0800	5.1	3	3	4	chloroplast
W9SG48	Chlorophyll a/b binding protein domain containing protein(predicted)	L484_023403	0.55	0.001639	Down	28.2790	13.8	3	3	3	chloroplast
W9RC20	Plastocyanin	L484_014304	0.55	0.001242	Down	16.5740	27.9	3	3	91	chloroplast
W9RTI2	ATP synthase subunit beta	L484_002144	0.55	0.000393	Down	40.7540	57.6	17	16	147	chloroplast
W9SVH5	Photosystem I reaction center subunit XI	L484_015127	0.53	0.004807	Down	23.1370	10.1	3	3	4	chloroplast
W9R4G6	Photosynthetic NDH subunit of	L484_006486	0.50	0.001945	Down	19.7270	5.2	1	1	1	chloroplast

	subcomplex B(predicted)										
W9RXN6	NADPH-protochlorophyllide oxidoreductase	L484_025670	0.50	0.001002	Down	43.0230	13.8	4	3	8	chloroplast
W9SMB1	Chlorophyll a-b binding protein, chloroplastic	L484_017723	0.48	0.001538	Down	29.5390	37.7	8	8	26	chloroplast
W9R7D8	Photosystem I reaction center subunit III	L484_025917	0.44	0.002206	Down	24.7260	19.8	4	4	14	chloroplast
W9R983	Chlorophyll a-b binding protein, chloroplastic	L484_002539	0.43	0.009359	Down	32.8640	3.4	1	1	1	chloroplast
W9QK62	Germin-like protein OS=Morus notabilis	L484_027371	0.43	0.000585	Down	22.1140	10.9	1	1	8	extracellular
W9RC44	Post-illumination chlorophyll fluorescence increase(predicted)	L484_025772	0.42	0.000750	Down	30.6170	13.4	3	3	11	chloroplast
W9R4W5	Chlorophyll a-b binding protein, chloroplastic	L484_002855	0.42	0.000188	Down	28.0730	51.3	7	1	36	chloroplast
W9SDQ4	Photosystem II D2 protein	L484_000839	0.41	0.002629	Down	34.0530	5.9	1	1	2	plasma membrane
W9R1X7	Chlorophyll a-b binding protein, chloroplastic	L484_006490	0.41	0.013522	Down	27.5560	6.8	1	1	2	chloroplast
W9SID3	Photosystem II 22 kDa protein, chloroplastic(predicted)	L484_004387	0.40	0.000059	Down	29.6220	15.4	4	4	9	chloroplast
W9SBB4	Chlorophyll a-b binding protein, chloroplastic	L484_024124	0.40	0.003051	Down	27.4540	13.2	3	3	9	chloroplast
W9RGN8	Chlorophyll a-b binding protein, chloroplastic	L484_018739	0.39	0.001298	Down	28.5320	13.3	3	3	7	chloroplast
W9QQ82	ATP synthase gamma chain	L484_013522	0.39	0.000947	Down	41.1970	24.3	8	8	22	chloroplast

W9QYC0	Chlorophyll a-b binding protein, chloroplastic	L484_026409	0.35	0.001186	Down	30.5470	33.8	7	4	17	chloroplast
W9QJH4	Photosystem II CP47 reaction center protein	L484_001781	0.34	0.000226	Down	50.6330	28.7	12	12	67	plasma membrane
W9QWB6	Photosystem II CP43 chlorophyll apoprotein	L484_024765	0.31	0.000280	Down	18.0550	35.2	7	7	39	chloroplast
W9SZU4	Chlorophyll a-b binding protein, chloroplastic	L484_020016	0.27	0.002296	Down	28.4650	18.5	5	2	28	chloroplast
W9S2Y9	Chlorophyll a-b binding protein, chloroplastic	L484_015572	0.27	0.001366	Down	31.4490	19.9	5	2	13	chloroplast
W9R5H7	Cytochrome b6	L484_001782	0.27	0.000353	Down	26.4060	10.7	2	2	10	chloroplast
W9SNV8	Photosystem II protein D1	L484_000029	0.24	0.000775	Down	34.7130	11	3	3	26	plasma membrane
W9S9S7	Chlorophyll a-b binding protein, chloroplastic	L484_012656	0.24	0.000903	Down	39.2790	30.6	8	8	36	chloroplast
W9R3W2	Photosystem II D2 protein(predicted)	L484_021838	0.24	0.002639	Down	8.1721	13.9	1	1	4	chloroplast
W9QNL6	Photosystem I P700 chlorophyll a apoprotein A1(predicted)	L484_010617	0.23	0.003068	Down	9.7429	14.1	1	1	4	nucleus
W9QRQ6	Photosystem Q(B) protein	L484_001783	0.14	0.001156	Down	17.4690	5.9	2	2	3	cytoplasm
**4. Carbohydrate metabolism (41)**											
W9RN59	Alpha-glucan water dikinase	L484_027728	8.73	0.000041	Up	160.7300	0.6	1	1	1	chloroplast
W9RTW2	Putative xyloglucan	L484_007462	4.74	0.000198	Up	35.5990	7.3	2	2	4	chloroplast
W9RMX9	Glucan endo-1,3-beta-glucosidase	L484_005596	4.63	0.000119	Up	36.9200	19.6	5	5	23	chloroplast
W9SKP3	Putative mannitol dehydrogenase	L484_026731	2.53	0.002941	Up	38.6210	2.8	1	1	1	chloroplast
W9RG14	Glucan endo-1,3-beta-glucosidase,	L484_004210	2.35	0.000201	Up	38.9120	21.6	7	7	14	chloroplast

	basic vacuolar isoform										
W9SX00	Putative beta-D-xylosidase 5	L484_003666	2.28	0.000385	Up	87.0010	11.2	8	4	19	vacuolar membrane
W9SN69	Putative beta-D-xylosidase 5	L484_000220	2.28	0.001366	Up	32.2270	20.5	5	1	15	extracellular
W9RY34	Alpha-galactosidase	L484_004288	2.09	0.000863	Up	41.9830	7.9	3	1	4	extracellular
W9S5E8	Phosphorylated carbohydrates phosphatase	L484_023341	2.01	0.006094	Up	27.7070	17.9	4	2	6	cytoplasm
W9R0K3	Lysosomal alpha-mannosidase	L484_008686	2.01	0.001084	Up	102.2900	12	11	11	32	nucleus
W9S2M8	Barwin-related endoglucanase(predicted)	L484_007512	1.98	0.000158	Up	23.2670	8.8	2	2	3	extracellular
W9RL71	Alpha-galactosidase	L484_012133	1.85	0.003578	Up	42.0890	7.4	3	2	3	chloroplast
W9SMB5	Glucan endo-1,3-beta-glucosidase 7	L484_027431	1.70	0.004207	Up	50.9850	9.8	5	4	18	chloroplast
W9QTY1	Pectate_lyase_3 domain-containing protein	L484_013735	1.67	0.002099	Up	26.0440	3.9	1	1	1	chloroplast
W9QW37	Alpha-L-fucosidase 2	L484_002039	1.65	0.000541	Up	74.9310	3	2	2	3	nucleus
W9RMQ8	UDP-glucose 4-epimerase	L484_002240	1.59	0.000085	Up	38.2310	14.1	4	4	6	cytoplasm
W9RKZ6	Outer membrane lipoprotein blc	L484_016874	2.30	0.001677	Up	21.3190	36.8	8	8	27	cytoplasm
W9SXA5	Beta-galactosidase	L484_008787	1.56	0.001935	Up	105.4800	14.3	12	12	24	cytoplasm
W9RIG7	Glycosyl hydrolase(predicted)	L484_018010	1.56	0.000250	Up	84.9420	3.2	2	2	3	chloroplast
W9R364	Lactoylglutathionelyase	L484_018049	1.56	0.001068	Up	33.1380	31.9	9	9	36	cytoplasm
W9S6V0	Putative aldolase-type TIM barrel(predicted)	L484_021704	1.52	0.002499	Up	32.2280	14.3	3	3	4	chloroplast
W9R689	Enolase(predicted)	L484_001983	1.50	0.000220	Up	45.1310	48.4	14	10	74	cytoplasm
W9RUZ9	Aldose 1-epimerase	L484_002001	0.67	0.004403	Down	39.6370	5.5	2	2	4	chloroplast
W9RT32	Putative Galactose-binding domain-containing	L484_003208	0.66	0.011378	Down	40.5780	4.8	2	2	2	chloroplast

	protein(predicted)										
W9RTC6	Glyceraldehyde-3-phosphate dehydrogenase	L484_006727	0.65	0.007871	Down	36.9170	41.3	13	4	44	cytoplasm
W9SFE6	HAD hydrolase, subfamily IA(predicted)	L484_000463	0.65	0.007678	Down	39.4500	9.6	3	3	8	chloroplast
W9RSF1	Pectinesterase	L484_011997	0.62	0.001171	Down	50.1610	16.4	6	2	41	chloroplast
W9QN56	Cytosolic endo-beta-N-acetylglucosaminidas e	L484_006939	0.61	0.000201	Down	77.1760	16	10	10	27	nucleus
W9RGS5	Transketolase	L484_025582	0.58	0.000085	Down	80.3050	29.9	19	19	117	chloroplast
W9QGQ4	Fructose-bisphosphate aldolase	L484_018536	0.57	0.000753	Down	42.3860	34.1	13	6	138	chloroplast
W9QNH5	Ribulose-phosphate 3-epimerase	L484_015956	0.56	0.006987	Down	30.1370	22.3	4	4	41	chloroplast
W9S0V2	6-phosphogluconate dehydrogenase, decarboxylating	L484_012090	0.54	0.003618	Down	54.2770	14.1	5	4	6	chloroplast
W9SAA4	UDP-glucose 6-dehydrogenase	L484_006653	0.52	0.003207	Down	52.3530	13.3	6	2	12	extracellular
W9R530	Beta-glucosidase 44	L484_017397	0.51	0.002320	Down	58.9260	14.1	8	8	12	cytoplasm
W9REN5	Sedoheptulose-1,7-bisphosphatase	L484_016237	0.51	0.001446	Down	42.6000	31.3	12	12	58	chloroplast
W9S3U8	Sucrose synthase	L484_019552	0.49	0.010307	Down	92.3860	7.9	6	4	15	cytoplasm
W9RPM3	Fructose-1,6-bisphosphatase	L484_023635	0.49	0.000789	Down	44.7530	24	7	7	22	chloroplast
W9RCJ5	probable galactinol--sucrose galactosyltransferase 2(predicted)	L484_017952	0.47	0.001596	Down	84.3000	13.9	8	8	11	chloroplast
W9R858	Hexosyltransferase OS=Morus notabilis	L484_026159	0.47	0.001293	Down	46.1450	11	4	4	7	cytoplasm
W9S6P4	Galactose-binding domain-like(predicted)	L484_021680	0.45	0.007431	Down	47.7990	1.8	1	1	2	cytoplasm
W9R4P1	UDP-glucose 6-dehydrogenase	L484_021004	0.32	0.002112	Down	53.0370	19	7	3	11	cytoplasm

5. Protein synthesis and degradation (118)

W9RIN5	40S ribosomal protein S29	L484_003677	2.15	0.000007	Up	9.5320	25.6	2	2	5	cytoplasm
W9QSD8	Proteasome subunit beta	L484_022430	1.83	0.020503	Up	24.5350	17.5	3	3	7	chloroplast
W9R470	UBA and UBX domain-containing protein	L484_004071	1.77	0.000611	Up	45.3260	2.9	1	1	2	nucleus
W9SCY9	Branched-chain-amino-acid aminotransferase	L484_007100	1.75	0.007131	Up	43.3720	12.7	4	3	9	chloroplast
W9R746	Aspartic proteinase Asp1	L484_013366	1.74	0.001909	Up	49.6100	3.3	2	1	4	nucleus
W9RHB5	Ubiquitin-conjugating enzyme E2 27	L484_008835	1.66	0.002792	Up	21.2790	11.3	2	2	3	cytoplasm
W9S1Y1	Kunitz-type serine protease inhibitor	L484_004398	1.66	0.002256	Up	26.7510	8.3	2	2	6	nucleus
W9SGM4	50S ribosomal protein L2	L484_022257	1.60	0.004081	Up	21.4400	12.5	2	2	13	nucleus
W9RZT7	40S ribosomal protein S26	L484_010677	1.59	0.002743	Up	16.8900	14.8	3	3	10	cytoplasm
W9RJZ5	Proteinase inhibitor	L484_005749	1.59	0.002126	Up	14.5420	14.6	2	2	8	chloroplast
W9QWL8	Small ubiquitin-related modifier	L484_000706	2.38	0.000091	Up	11.1560	11.9	2	2	15	nucleus
W9SCT8	Aspartic proteinase-like protein 1	L484_026627	2.03	0.003046	Up	102.3800	1.3	1	1	2	chloroplast
W9RZU9	Non-specific serine/threonine protein kinase	L484_010692	2.00	0.028518	Up	55.8640	2.9	2	2	2	cytoplasm
W9RFB0	Alpha/Beta hydrolase fold containing protein(predicted)	L484_018635	2.00	0.000077	Up	44.8430	7	2	2	5	chloroplast
W9R883	Heparan-alpha-glucosaminide N-acetyltransferase-like(predicted)	L484_022569	1.58	0.009062	Up	13.7400	15.9	2	2	4	mitochondria
W9QGF9	Subtilisin-like protease	L484_018355	1.53	0.003638	Up	85.2650	5.8	4	3	9	endoplasmic reticulum
W9R0T3	Lysosomal alpha-mannosidase	L484_008682	1.53	0.000999	Up	100.4900	9.9	7	7	20	nucleus

W9T1B7	Ribosomal protein L22p/L17e family protein isoform 1(predicted)	L484_001651	1.53	0.008050	Up	11.2470	27.2	2	2	4	nucleus
W9R795	Mitochondrial glycoprotein(predicted)	L484_012583	1.53	0.003358	Up	29.0920	8.1	2	2	2	mitochondria
W9QJN7	Mitochondrial glycoprotein(predicted)	L484_014460	1.79	0.014082	Up	28.1550	3.7	1	1	2	mitochondria
W9S9U6	Epidermis-specific secreted glycoprotein EP1	L484_018658	1.59	0.001072	Up	48.2760	11.1	5	2	6	extracellular
W9QG40	Acetyltransferase component of pyruvate dehydrogenase complex	L484_003223	1.53	0.002047	Up	55.9520	14.5	7	7	16	mitochondria
W9QLC8	Pyruvate dehydrogenase E1 component subunit beta	L484_017496	1.52	0.010891	Up	32.0360	17	4	4	13	chloroplast
W9R0R6	NADH dehydrogenase [ubiquinone] iron-sulfur protein 3	L484_004153	1.52	0.014693	Up	10.1510	14.9	1	1	4	nucleus
W9SAU4	Alpha-galactosidase	L484_011230	1.52	0.000276	Up	45.9300	10.7	5	5	9	chloroplast
W9RUB7	Alpha-galactosidase	L484_023052	1.52	0.003583	Up	47.4950	17.3	6	4	11	chloroplast
W9R0F0	Putative E3 ubiquitin-protein ligase HERC1	L484_023954	1.52	0.000870	Up	115.1300	0.9	1	1	2	nucleus
W9QQ03	60S ribosomal protein L23	L484_005704	0.67	0.001709	Down	15.0270	62.9	7	7	22	cytoplasm
W9S7C8	Aspartic proteinase nepenthesin-2	L484_007149	0.67	0.003998	Down	51.7340	9.4	4	4	9	extracellular
W9R6D5	Subtilisin-like protease	L484_027403	0.67	0.006134	Down	80.8410	3.2	2	2	2	chloroplast
W9S9Z2	Serine/threonine protein phosphatase(predicted)	L484_002545	0.66	0.015836	Down	15.4400	8.1	1	1	1	nucleus
W9QQR2	Ubiquitin-activating enzyme E1 2	L484_011695	0.66	0.003708	Down	121.2700	11.8	11	11	18	chloroplast
W9RSX6	60S ribosomal protein L11-2	L484_021351	0.66	0.018707	Down	23.3580	19.3	4	4	9	cytoplasm
W9QC04	60S ribosomal protein L18a	L484_009910	0.66	0.018304	Down	32.2630	3.1	1	1	3	chloroplast

W9R9J3	Acylamino-acid-releasing enzyme	L484_010277	0.65	0.006952	Down	28.0250	11.5	2	2	2	chloroplast
W9R6G5	60S ribosomal protein L12	L484_007651	0.65	0.000924	Down	17.8520	50	7	7	22	cytoplasm
W9QLJ3	Pyruvate dehydrogenase E1 component subunit beta	L484_017497	1.58	0.009495	Up	16.5020	22.7	3	3	17	cytoplasm
W9SV80	Pyruvate dehydrogenase E1 component subunit beta	L484_021646	0.64	0.023800	Down	44.1000	2.5	1	1	2	chloroplast
W9RD86	50S ribosomal protein L6	L484_001985	0.64	0.007524	Down	24.9850	27.1	6	6	12	chloroplast
W9QVZ3	40S ribosomal protein S2-4	L484_005567	0.64	0.002309	Down	29.8370	29.2	9	9	19	endoplasmic reticulum
W9SG16	Subtilisin-like protease	L484_010262	0.64	0.000522	Down	81.2220	10	6	6	7	chloroplast
W9SN88	Histidine--tRNA ligase	L484_000283	0.63	0.007016	Down	65.0190	1.9	1	1	1	cytoplasm
W9S6R7	Clp protease-related protein	L484_023195	0.63	0.025563	Down	30.5810	6.9	2	2	4	chloroplast
W9QLF2	60S ribosomal protein L31	L484_005476	0.63	0.013813	Down	13.7860	6.7	1	1	6	cytoplasm
W9RR53	60S ribosomal protein L7-4	L484_003162	0.63	0.005285	Down	33.9670	30.9	10	2	23	cytoplasm
W9QMR9	Acetyl-CoA acetyltransferase, cytosolic 1	L484_004867	0.62	0.002375	Down	40.8480	2	1	1	2	chloroplast
W9SIT5	50S ribosomal protein L2	L484_008964	0.62	0.001969	Down	14.8400	20.3	2	2	4	cytoplasm
W9SDB5	Ribosomal protein L19	L484_005240	0.62	0.002298	Down	24.0700	26.7	5	5	11	nucleus
W9RKB9	Ribosomal protein	L484_007939	0.62	0.001605	Down	39.8220	26.5	9	9	26	chloroplast
W9RXF3	Ribosomal protein	L484_014638	0.62	0.000112	Down	30.2860	17.2	4	4	10	cytoplasm
W9R236	Elongation factor G, chloroplastic	L484_019003	0.61	0.003598	Down	86.6200	13.6	9	9	18	mitochondria
W9QMK6	Translation initiation factor IF-3	L484_013954	0.62	0.013720	Down	33.2550	11.5	3	3	5	chloroplast
W9S2S7	60S ribosomal protein L4	L484_003849	0.61	0.000061	Down	44.9580	32.4	12	12	32	cytoplasm
W9S431	50S ribosomal protein L31	L484_021939	0.60	0.002797	Down	14.8680	60.2	7	7	16	chloroplast
W9QSW1	40S ribosomal protein S24	L484_006178	0.60	0.003257	Down	18.5100	15	2	2	6	cytoplasm
W9T0E9	Putative proline--tRNA ligase	L484_022724	0.60	0.000009	Down	57.6500	8.9	4	4	5	cytoplasm

W9R7K2	Metal-dependent protein hydrolase(predicted)	L484_001701	0.60	0.006795	Down	43.2810	17.1	6	6	8	chloroplast
W9RI49	40S ribosomal protein S15 OS=Morus notabilis	L484_024561	0.60	0.004498	Down	17.2420	36.8	5	5	31	cytoplasm
W9QQ56	40S ribosomal protein S9-2 OS=Morus notabilis	L484_005300	0.59	0.000276	Down	31.2400	24.7	8	8	18	nucleus
W9RID5	Serine/threonine-protein kinase SAPK2	L484_012739	0.59	0.000501	Down	67.1380	8.6	6	5	14	nucleus
W9RK49	Lysosomal Pro-X carboxypeptidase	L484_014075	0.59	0.002062	Down	52.4880	6	3	3	5	chloroplast
W9S2Y6	Proline--tRNA ligase	L484_014247	0.59	0.002018	Down	65.5810	4.6	3	3	3	cytoplasm
W9R6V6	60S ribosomal protein L18-2	L484_001926	0.58	0.009763	Down	22.0220	21.2	4	2	6	cytoplasm
W9RLY1	Putative receptor-like protein kinase	L484_009296	0.58	0.007178	Down	93.6780	0.9	1	1	1	plasma membrane
W9S4K0	Metalloendoproteinase 1	L484_016825	0.57	0.000869	Down	37.5430	7.5	2	2	2	chloroplast
W9RFD6	30S ribosomal protein S11	L484_012144	0.57	0.000524	Down	16.0180	16.2	2	2	2	cytoplasm
W9RHR9	ATP-dependent zinc metalloprotease FTSH 2	L484_021363	0.56	0.002585	Down	74.2420	31.8	17	17	42	chloroplast
W9RWK4	26S proteasome non-ATPase regulatory subunit RPN12A	L484_007834	0.56	0.001039	Down	41.7690	10.9	3	3	3	chloroplast
W9RMB6	60S ribosomal protein L17-2	L484_005498	0.55	0.000167	Down	22.8010	16.3	3	1	6	nucleus
W9R644	60S acidic ribosomal protein P0	L484_026391	0.54	0.000493	Down	34.1500	14.1	5	5	13	cytoplasm
W9RRF5	40S ribosomal protein S3-3	L484_002613	0.54	0.000884	Down	31.7360	35.8	10	10	21	cytoplasm
W9RUQ0	60S ribosomal protein L26-1	L484_009396	0.54	0.000048	Down	16.6530	36.3	6	6	13	cytoplasm
W9RF78	60S ribosomal protein L5	L484_020380	0.53	0.002222	Down	34.9240	21.6	8	8	27	cytoplasm
W9RT39	Nascent polypeptide-associated complex subunit alpha-like protein	L484_002597	0.53	0.003336	Down	24.4760	24.2	4	4	16	cytoplasm

	2										
W9S7X2	40S ribosomal protein S23	L484_024448	0.53	0.000650	Down	19.9530	11.2	3	3	6	cytoplasm
W9S011	40S ribosomal protein S11	L484_008524	0.52	0.002924	Down	20.2820	15.2	3	3	8	chloroplast
W9RLX2	60S ribosomal protein L10	L484_024157	0.52	0.006019	Down	64.7650	6.6	4	4	10	chloroplast
W9SH70	2-phosphoglycolate phosphatase(predicted)	L484_020641	0.51	0.000995	Down	40.2170	30.3	9	9	17	chloroplast
W9S425	Pyruvate dehydrogenase E1 component subunit alpha	L484_019631	0.51	0.002504	Down	47.6720	4.9	2	2	4	chloroplast
W9RUW3	30S ribosomal protein S17	L484_026214	0.51	0.001228	Down	18.3120	17.4	4	4	8	chloroplast
W9QJ02	60S ribosomal protein L6	L484_013925	0.50	0.000044	Down	40.8950	12.8	5	2	13	chloroplast
W9SAM0	50S ribosomal protein L27	L484_010778	0.50	0.002447	Down	21.4360	16.3	3	3	4	chloroplast
W9R517	50S ribosomal protein L3-1	L484_023807	0.49	0.000274	Down	29.9490	37.9	9	9	19	chloroplast
W9RXW8	60S ribosomal protein L13a-4	L484_021592	0.48	0.000572	Down	30.9760	9.6	3	3	5	chloroplast
W9RZ68	30S ribosomal protein S12-B	L484_018650	0.48	0.019528	Down	17.1710	5.7	1	1	2	cytoplasm
W9RCG2	Multiple organellar RNA editing factor(predicted)	L484_021132	0.47	0.000343	Down	27.3930	7	1	1	1	chloroplast
W9SH92	40S ribosomal protein S14-3	L484_021684	0.47	0.000708	Down	30.5300	16.2	3	1	10	cytoplasm
W9RI64	Mediator of RNA polymerase II transcription subunit 14	L484_024576	0.47	0.002682	Down	239.8100	3.6	7	7	13	cytoplasm
W9S1B4	Nucleolar and coiled-body phosphoprotein 1-like(predicted)	L484_027628	0.47	0.004245	Down	139.7900	0.8	1	1	3	nucleus
W9S7Q1	29 kDa ribonucleoprotein B	L484_024380	0.47	0.001771	Down	31.1970	44.9	8	8	19	chloroplast
W9S2P4	Eukaryotic translation initiation factor 5A	L484_015229	0.47	0.001437	Down	17.4800	52.5	8	5	29	cytoplasm
W9RTX6	Ribosomal_L18e/L15P domain-containing protein	L484_000089	0.47	0.003330	Down	16.4400	30.8	4	4	10	cytoplasm

W9S6X5	30S ribosomal protein S5	L484_014494	0.47	0.001682	Down	36.2190	29.1	10	10	28	chloroplast
W9QKG7	40S ribosomal protein S3a	L484_006165	0.46	0.000030	Down	30.0530	21	5	5	8	cytoplasm
W9RLN2	50S ribosomal protein L15	L484_014644	0.46	0.004171	Down	13.3180	37.9	6	6	12	nucleus
W9S148	30S ribosomal protein S9	L484_021241	0.45	0.001845	Down	14.8510	17.3	2	2	5	chloroplast
W9RRS6	60S ribosomal protein L34	L484_005014	0.45	0.000398	Down	13.7100	23.3	3	3	6	cytoplasm
W9R491	40S ribosomal protein S8	L484_009528	0.44	0.000210	Down	24.0860	19.5	4	4	6	nucleus
W9S0D7	Eukaryotic translation initiation factor 5A-4	L484_015725	0.44	0.000322	Down	23.2040	29.1	5	1	10	chloroplast
W9SA24	60S ribosomal protein L36	L484_006513	0.42	0.001669	Down	20.0160	16.9	3	3	4	cytoplasm
W9R7J6	40S ribosomal protein S7	L484_019950	0.41	0.000173	Down	21.9750	17.8	2	2	3	cytoplasm
W9QZ03	Putative receptor-like protein kinase	L484_013394	0.39	0.000952	Down	68.8690	1.3	1	1	1	plasma membrane
W9QLN7	Ribosomal RNA small subunit methyltransferase G(predicted)	L484_012399	0.39	0.000287	Down	18.5990	11.7	2	2	4	cytoplasm
W9R7M9	30S ribosomal protein S4, chloroplastic	rps4	0.38	0.000586	Down	23.3630	16.9	3	3	6	chloroplast
W9S5Y9	60S ribosomal protein L3	L484_004471	0.38	0.000182	Down	46.9150	29.3	12	12	37	cytoplasm
W9QKR4	50S ribosomal protein L29	L484_022129	0.38	0.001302	Down	47.7090	9.9	4	4	8	nucleus
W9RBT1	30S ribosomal protein S2	L484_013124	0.37	0.000345	Down	24.2970	3.8	1	1	1	cytoplasm
W9RJ94	60S ribosomal protein L13-2	L484_004330	0.36	0.001851	Down	23.7780	22.7	5	5	10	chloroplast
W9RN06	Elongation factor 1-gamma	L484_011642	0.35	0.009763	Down	52.2260	1.5	1	1	3	chloroplast
W9QZE3	Eukaryotic initiation factor 4A-8	L484_013471	0.35	0.001688	Down	48.0390	25.8	11	1	38	cytoplasm
W9RFX5	50S ribosomal protein L13	L484_017521	0.32	0.001162	Down	28.0830	9.6	3	3	7	chloroplast
W9S021	50S ribosomal protein L4	L484_022055	0.30	0.001353	Down	32.0730	19.1	5	5	12	chloroplast
W9SIL9	60S ribosomal protein L7a	L484_010806	0.29	0.001420	Down	33.0550	9.3	3	3	5	chloroplast
W9S0F4	60S ribosomal protein L17-2	L484_005471	0.29	0.000736	Down	35.6760	7.6	3	1	8	nucleus

W9R894	50S ribosomal protein L21	L484_006839	0.28	0.002677	Down	25.5360	6.4	2	2	4	chloroplast
W9QQL7	50S ribosomal protein L24	L484_021161	0.26	0.000228	Down	21.3000	6.2	2	2	4	chloroplast
W9RMX2	60S ribosomal protein L38	L484_005586	0.26	0.003193	Down	17.6780	8.3	2	2	2	mitochondria
W9S1K3	Putative ribosomal protein S20(predicted)	L484_009619	0.26	0.001234	Down	19.7400	11.1	2	2	7	chloroplast
**6. Signal transduction (28)**											
W9QQG6	Calcium-dependent protein kinase 9	L484_017849	2.11	0.002348	Up	62.1860	2.9	1	1	1	nucleus
W9SA09	ABSCISIC ACID-INSENSITIVE 5-like protein 5	L484_009555	1.87	0.003521	Up	47.3140	14.8	6	6	8	nucleus
W9QIF0	LRR receptor-like serine/threonine-protein kinase RPK2	L484_021029	1.86	0.001689	Up	125.0700	7.2	8	8	10	plasma membrane
W9S7I0	Remorin(predicted)	L484_015780	1.85	0.000953	Up	22.1230	13.6	3	2	22	nucleus
W9QYD5	DnaJ homolog subfamily B member 13	L484_008254	1.79	0.006666	Up	37.3530	5.9	2	2	2	cytoskeleton
W9SPI8	Endosulphine(predicted)	L484_015675	1.78	0.001508	Up	17.3130	11.9	2	2	4	nucleus
W9SH04	Profilin	L484_006986	1.70	0.001563	Up	151.0200	2.5	2	2	7	plasma membrane
W9QZH0	PTB domain-containing protein	L484_015993	1.65	0.005122	Up	43.0750	7.3	3	3	4	nucleus
W9S3I5	HIT domain-containing protein	L484_018496	1.63	0.001913	Up	18.6260	30.1	4	4	13	cytoplasm
W9RC16	Serine/threonine protein kinase(predicted)	L484_014298	1.58	0.000929	Up	59.0840	4.5	2	2	2	extracellular
W9R7Z9	GRAM domain-containing protein	L484_026039	1.57	0.007457	Up	33.1740	3	1	1	4	nucleus
W9R3F4	Histidine-containing phosphotransfer protein 1	L484_008998	1.56	0.000010	Up	23.0390	5.3	1	1	3	nucleus

W9S785	auxin-repressed 12.5 kDa protein(predicted)	L484_015082	1.56	0.000950	Up	13.3630	50	5	5	18	nucleus
W9QXZ9	Clathrin light chain	L484_026403	1.56	0.020233	Up	30.6990	5.4	2	2	6	nucleus
W9QR00	Caspase-like domain containing protein(predicted)	L484_019225	1.55	0.002630	Up	47.1950	12	4	4	9	cytoplasm
W9SFA4	Nucleoside diphosphate kinase	L484_006917	1.51	0.002558	Up	25.8440	23.4	5	5	19	chloroplast
W9SGS5	Cyclic phosphodiesterase-like protein 2(predicted)	L484_008392	0.65	0.005018	Down	20.9580	10.2	1	1	2	nucleus
W9SFL0	GTP-binding nuclear protein	L484_005609	0.64	0.003409	Down	63.6440	14.3	8	2	21	chloroplast
W9RPA1	Preprotein translocase subunit SECE1	L484_005416	0.63	0.014570	Down	18.6340	6.5	1	1	3	chloroplast
W9RXJ7	14-3-3 protein 4	L484_008661	0.62	0.004571	Down	81.3680	16.8	10	6	33	chloroplast
W9RF74	Serine/threonine-protein kinase KIPK	L484_008996	0.61	0.001413	Down	91.4650	1.3	1	1	1	nucleus
W9SI26	Adenosine kinase 2	L484_013051	0.55	0.002932	Down	37.4780	22.3	6	6	17	cytoplasm
W9SQH3	Ras-related protein RABA2a	L484_000240	0.55	0.006033	Down	18.8300	14.6	2	1	6	nucleus
W9QR64	Peptide-methionine (R)-S-oxide reductase	L484_010429	0.54	0.003532	Down	23.9940	10.4	2	2	6	chloroplast
W9R640	5-methyltetrahydropteroyltriglutamate--homocysteine methyltransferase	L484_009365	0.54	0.000814	Down	84.6140	20.4	14	10	36	cytoplasm
W9R7X5	14-3-3-like protein	L484_012741	0.50	0.003401	Down	29.1180	49.4	11	7	34	plasma membrane
W9SIJ9	WD repeat-containing protein 44	L484_021300	0.38	0.001609	Down	103.2100	0.7	1	1	4	nucleus
W9SBC2	WD repeat-containing protein 82-B	L484_020552	1.89	0.021760	Up	37.1560	3.6	1	1	1	cytoplasm

7. Membrane transport (27)

W9RHB0	Mitochondrial import inner membrane translocase subunit	L484_008830	1.75	0.010760	Up	8.8982	52.6	4	4	12	chloroplast
W9R7Y6	Putative GPI-anchored protein	L484_002667	1.67	0.004782	Up	18.8460	6.2	1	1	1	chloroplast
W9RGK0	VPS37 C-terminal domain-containing protein	L484_022617	1.63	0.002371	Up	26.5510	4.3	1	1	1	nucleus
W9QNM0	Putative ADP-ribosylation factor GTPase-activating protein AGD13	L484_010622	1.59	0.003194	Up	18.7910	20.1	3	3	7	plasma membrane
W9SHG8	Outer envelope pore protein 24A(predicted)	L484_021189	1.59	0.001251	Up	23.3230	36.2	7	7	10	cytoplasm
W9QZI8	Protein transport protein SEC23	L484_016334	3.10	0.000064	Up	79.8640	1.9	1	1	1	nucleus
W9RPG2	Polyol transporter 5	L484_026936	2.70	0.001424	Up	58.7250	1.3	1	1	1	plasma membrane
W9QWS6	Translocator-like protein	L484_009090	2.43	0.001640	Up	21.4890	10.9	2	2	2	vacuolar membrane
W9SDJ5	Cationic amino acid transporter 6	L484_023396	2.06	0.004993	Up	62.1090	1.4	1	1	1	plasma membrane
W9RP61	Golgin family A protein(predicted)	L484_012452	1.98	0.000834	Up	23.0260	8.5	1	1	1	nucleus
W9R8X9	Mitochondrial pyruvate carrier	L484_012453	1.52	0.005794	Up	45.5300	13.3	5	5	11	nucleus
W9R3Y8	Pyrophosphate-energized vacuolar membrane proton pump	L484_025732	0.65	0.002872	Down	80.4800	5.5	5	5	9	vacuolar membrane
W9RB82	V-type proton ATPase subunit C	L484_008700	0.65	0.000110	Down	43.6840	10.9	4	4	9	chloroplast
W9RZQ2	Transmembrane 9 superfamily member	L484_019271	0.64	0.015546	Down	67.8060	1.9	1	1	1	plasma membrane
W9SDS9	Coatomer subunit alpha	L484_008431	0.61	0.001606	Down	136.4300	4.1	5	5	6	chloroplast
W9SZA5	Plasma membrane ATPase	L484_010085	0.60	0.002548	Down	104.7600	21.6	19	10	46	plasma membrane

W9SZF7	mitochondrial dicarboxylate/tricarboxylate transporter DTC-like(predicted)	L484_019087	0.58	0.001291	Down	31.9590	14.7	4	4	9	cytoplasm
W9R938	Putative voltage-gated potassium channel subunit beta	L484_017907	0.58	0.004174	Down	44.6860	6	3	2	10	chloroplast
W9S238	V-type proton ATPase catalytic subunit A	L484_003800	0.54	0.000766	Down	68.7520	26.6	14	14	29	chloroplast
W9RMA5	Dynamin-related protein 5A	L484_002239	0.53	0.011814	Down	68.3260	7	4	4	4	cytoplasm
W9S019	Transmembrane protein(predicted)	L484_020061	0.52	0.001296	Down	27.0950	10.5	2	2	3	plasma membrane
W9R2K8	Coatomer subunit beta'-2	L484_020834	0.51	0.000089	Down	115.6900	2.3	2	1	2	chloroplast
W9QDE6	Importin N-terminal domain-containing protein	L484_019696	0.49	0.002003	Down	128.9000	3.7	3	3	4	cytoplasm
W9R165	Coatomer subunit gamma	L484_026915	0.48	0.040216	Down	95.4620	1.5	1	1	1	chloroplast
W9S6K8	Coatomer subunit beta	L484_004976	0.48	0.000373	Down	106.4100	0.8	1	1	1	nucleus
W9SBM6	Coatomer subunit delta	L484_018509	0.48	0.003198	Down	57.8310	13.6	6	6	8	chloroplast
W9QCA7	PRA1 family protein	L484_012274	0.43	0.008398	Down	22.8960	4.2	1	1	1	chloroplast
**8. Transcription regulation (32)**											
W9RGH7	Heme-binding-like protein, chloroplastic(predicted)	L484_023627	2.12	0.000337	Up	22.2200	28.5	6	6	16	cytoplasm
W9QFK9	Nuclear transcription factor Y subunit B-3	L484_018224	1.95	0.009116	Up	21.5790	4.3	1	1	2	nucleus
W9R1Z8	Transcription factor HBP-1a	L484_007879	1.95	0.001305	Up	42.8770	7.7	2	2	4	nucleus
W9S077	Transcription factor VIP1	L484_017885	1.93	0.001636	Up	37.0180	11.2	4	4	9	nucleus
W9S6J5	NAC domain-containing protein 68	L484_005670	1.89	0.005565	Up	28.8850	3.2	1	1	1	cytoplasm

W9R561	Transcription termination and cleavage factor(predicted)	L484_019959	1.88	0.008347	Up	42.4870	2.8	1	1	1	nucleus
W9RA09	Methyl-CpG-binding domain-containing protein 10	L484_006426	1.82	0.000031	Up	32.7100	27.4	8	7	31	nucleus
W9QYF0	DYW_deaminase domain-containing protein	L484_014600	1.81	0.000509	Up	66.6060	1.4	1	1	4	cytoplasm
W9R6Z7	RNA-binding protein 24	L484_024805	1.77	0.001151	Up	30.9990	7	2	1	2	nucleus
W9SGD0	Flowering time control protein FCA	L484_004575	1.70	0.005570	Up	111.3600	2.1	2	2	2	plasma membrane
W9RQX1	Polyadenylate-binding protein RBP45B	L484_012860	1.60	0.000154	Up	43.9870	5.5	2	2	6	nucleus
W9QUV7	Transcription regulator AsnC-type(predicted)	L484_027359	1.57	0.007965	Up	23.8370	13	2	2	3	cytoplasm
W9QJ86	Transcription initiation factor TFIID subunit 4B	L484_022370	1.54	0.007436	Up	105.0900	1.7	1	1	2	nucleus
W9RB21	Sm domain-containing protein OS=Morus notabilis	L484_019349	1.54	0.001490	Up	30.3590	13.7	3	3	16	chloroplast
W9S209	Heterogeneous nuclear ribonucleoprotein 27C	L484_014869	1.53	0.003741	Up	36.5220	21.6	6	6	11	nucleus
W9R3G4	Far upstream element-binding protein 2	L484_019523	1.51	0.002181	Up	71.5210	17.3	11	11	51	nucleus
W9RY62	Elongation factor 1-delta	L484_005497	0.66	0.004717	Down	26.1370	38.6	9	8	27	cytoplasm
W9SBM3	Peptide chain release factor 1	L484_020489	0.65	0.006474	Down	46.6500	6.5	2	2	3	chloroplast
W9RUU2	Histone H2A	L484_004666	0.64	0.024099	Down	14.6870	23.9	3	2	17	nucleus
W9R7J8	Glycine-rich RNA-binding protein 2	L484_002067	0.64	0.004823	Down	15.5450	19.2	2	2	4	chloroplast

W9R4M1	Dimethylallyl, adenosine tRNA methylthiotransferase(predicted)	L484_018998	0.64	0.019961	Down	67.4090	7.2	4	4	12	chloroplast
W9S0W0	DEAD-box ATP-dependent RNA helicase 8	L484_017319	0.58	0.001816	Down	49.6510	5.5	2	2	3	cytoplasm
W9REH8	DEAD-box ATP-dependent RNA helicase 37	L484_007324	1.68	0.030763	Up	66.2960	6.7	3	3	3	nucleus
W9QZM0	Histone H4	L484_001850	0.56	0.000410	Down	11.4090	57.3	7	7	31	nucleus
W9R8Q4	NAD(P)-bd_dom domain-containing protein	L484_013631	0.55	0.000648	Down	53.9940	9.7	5	5	7	chloroplast
W9RCR1	Nucleic acid-binding, OB-fold containing protein(predicted)	L484_027775	0.55	0.001862	Down	17.6610	19	2	2	5	cytoplasm
W9QT90	RNA-binding protein Nova-1	L484_007994	0.55	0.001382	Down	37.2780	9.5	3	3	3	nucleus
W9S3D3	Eukaryotic translation initiation factor 5A	L484_013212	0.55	0.000739	Down	17.4690	51.9	8	3	16	chloroplast
W9SI90	Phenylalanine--tRNA ligase	L484_014974	0.54	0.002421	Down	50.6700	4.5	2	2	4	chloroplast
W9QUQ5	Eukaryotic initiation factor 4A-15	L484_027308	0.51	0.000218	Down	95.2360	14.9	12	2	43	nucleus
W9RYZ9	Histone H3	L484_019397	0.49	0.001556	Down	15.4060	28.7	5	5	13	nucleus
W9RMA1	Histone	L484_005755	0.48	0.000416	Down	29.5430	9.5	3	3	4	nucleus
**9. Phytohormone biosynthesis and responses (12)**											
W9RNE2	Phospholipase A1-IIdelta	L484_010361	3.42	0.000278	Up	48.5500	7.2	4	3	8	cytoplasm
W9SLR9	1-aminocyclopropane-1-carboxylate oxidase-1-like protein	L484_004433	2.15	0.003708	Up	56.5740	1.9	1	1	3	cytoplasm
W9QFG3	IAA-amino acid hydrolase ILR1-like 5	L484_008123	2.06	0.000884	Up	47.4520	18.7	6	6	13	extracellular
W9QLX8	Small rubber particle	L484_009092	1.69	0.010798	Up	26.0540	7.2	1	1	1	chloroplast

	protein(predicted)										
W9SR96	Tryptophan aminotransferase-related protein 4	L484_001340	1.68	0.006129	Up	53.4320	15	7	7	8	vacuolar membrane
W9R5S0	Chaperone protein dnaJ 8	L484_018075	1.52	0.012604	Up	17.8540	7	1	1	1	cytoplasm
W9RPN7	Maternal effect embryo arrest(predicted)	L484_027009	1.51	0.006075	Up	23.2930	13.9	3	3	5	mitochondria
W9R766	ATP-dependent zinc metalloprotease FTSH 2	L484_024890	0.63	0.000598	Down	76.1380	25.2	13	13	34	chloroplast
W9SFC7	Auxin-responsive protein	L484_011267	0.62	0.003260	Down	43.7900	5	2	1	6	nucleus
W9QYS6	Anti-muellerian hormone type-2 receptor(predicted)	L484_014606	0.55	0.004070	Down	17.4400	7.7	1	1	2	chloroplast
W9RZ60	Phospholipase A1-IIdelta	L484_010360	0.38	0.001237	Down	50.4090	9.7	4	3	9	cytoplasm
W9QF93	GrpE protein homolog	L484_004151	0.37	0.005567	Down	57.2200	6.8	3	3	5	nucleus
**10. Lipid metabolism (19)**											
W9RSQ3	Acetyl-coenzyme A carboxylase carboxyl transferase subunit alpha	L484_017321	2.41	0.000922	Up	101.5100	0.8	1	1	1	cytoplasm
W9QX55	Lipoxygenase	L484_001068	2.36	0.000305	Up	87.0220	6.7	6	4	15	cytoplasm
W9QUU1	Non-specific lipid-transfer protein	L484_002202	2.21	0.016681	Up	12.1020	13.2	2	2	25	extracellular
W9QQR7	AAI domain-containing protein	L484_016938	1.89	0.000244	Up	11.9330	10.4	1	1	1	extracellular
W9RJG5	Lipoxygenase	L484_000759	1.70	0.000098	Up	100.5600	7.1	6	4	11	cytoplasm
W9RJR4	3-ketoacyl-CoA thiolase 2	L484_017921	1.64	0.001464	Up	48.6660	23.1	9	9	21	cytoplasm
W9SF71	Acylpyruvase FAHD1	L484_007204	1.61	0.002284	Up	23.9400	17.2	4	4	10	cytoplasm
W9R965	Plastid-lipid-associated protein	L484_014140	2.37	0.000970	Up	34.9870	13	5	5	21	chloroplast
W9RPE6	GDSL esterase/lipase 1	L484_005045	1.56	0.000658	Up	41.8570	2.9	1	1	2	nucleus
W9QQB9	Apolipoprotein D	L484_017803	1.50	0.002799	Up	37.6600	32.9	9	9	36	chloroplast
W9RQ03	Acyl-coenzyme A oxidase	L484_024072	0.66	0.047574	Down	74.8660	6.2	5	5	9	cytoplasm

W9SL29	Lipoxygenase(predicted)	L484_000135	0.65	0.002279	Down	7.9270	46.8	4	4	7	cytoplasm
W9QTZ7	Epoxide hydrolase 2	L484_011047	0.64	0.004894	Down	35.4450	14	4	4	7	cytoplasm
W9RV50	GDSL esterase/lipase APG	L484_026305	0.64	0.021788	Down	38.0020	13.6	4	4	7	extracellular
W9S3Z1	ATP-citrate synthase beta chain protein 1	L484_009247	0.64	0.003420	Down	66.0350	7.6	3	3	5	chloroplast
W9QMU5	Putative plastid-lipid-associated protein 12	L484_018429	0.64	0.016932	Down	44.0260	5.6	2	2	2	chloroplast
W9R031	Lipoxygenase	L484_007985	0.63	0.002991	Down	96.0410	17	12	10	24	cytoplasm
W9QY99	Lipoxygenase	L484_018382	0.48	0.000247	Down	104.0700	9	8	6	13	chloroplast
W9SFQ0	Putative carboxylesterase 8	L484_027948	0.38	0.002275	Down	38.0130	17.2	5	5	8	nucleus
**11. Nitrogen assimilation and amino acid metabolism (20)**											
W9S9M7	Glutamate dehydrogenase	L484_017087	1.62	0.000355	Up	44.3030	11.7	4	3	7	mitochondria
W9RSM5	Protein-L-isoaspartate O-methyltransferase	L484_018948	1.61	0.004439	Up	41.2340	13.5	5	5	14	chloroplast
W9R093	Glutamate dehydrogenase	L484_014749	1.58	0.001878	Up	44.1780	12.4	4	3	10	cytoplasm
W9RP10	Aspartokinase	L484_008693	5.59	0.002011	Up	60.3830	2.2	1	1	2	chloroplast
W9RJK9	Asparagine synthetase [glutamine-hydrolyzing]	L484_007597	2.57	0.000009	Up	64.4250	11.4	7	4	15	cytoplasm
W9R093	Glutamate dehydrogenase	L484_014749	1.58	0.001878	Up	44.1780	12.4	4	3	10	cytoplasm
W9R3V0	N-acyl-L-amino-acid amidohydrolase(predicted)	L484_002939	1.51	0.001870	Up	52.1620	13.4	6	5	16	chloroplast
W9RZN3	Serine hydroxymethyltransferase	L484_025550	0.62	0.001721	Down	39.1390	18.8	5	5	25	mitochondria
W9S1K2	Argininosuccinate synthase	L484_002180	0.59	0.000410	Down	102.2000	6.3	4	4	5	cytoplasm
W9RY59	Ferredoxin--nitrite reductase	L484_027526	0.52	0.001786	Down	65.2120	16	8	8	13	chloroplast
W9QYB1	Methionine aminotransferase	L484_020169	0.51	0.004262	Down	50.5810	4.8	2	2	4	mitochondria

W9QW05	Serine--glyoxylate aminotransferase	L484_002102	0.49	0.000181	Down	39.6420	44.1	11	11	40	cytoplasm
W9S915	Ketol-acid reductoisomerase	L484_027131	0.49	0.000043	Down	57.7390	8.9	4	3	8	cytoplasm
W9QCD4	Uroporphyrinogen decarboxylase	L484_012310	0.46	0.004409	Down	26.3390	13.9	2	2	4	chloroplast
W9REF8	Glutamate decarboxylase	L484_007798	0.44	0.001619	Down	41.6490	8.8	3	3	7	cytoplasm
W9R5W4	Adenosylhomocysteinase	L484_026840	0.44	0.000550	Down	53.3790	41.2	17	17	47	cytoplasm
W9R532	Guanine nucleotide-binding protein subunit beta-like protein	L484_017402	0.42	0.001751	Down	36.1750	20.6	6	6	13	chloroplast
W9RGU6	Lysine-specific demethylase 2A-like(predicted)	L484_026189	0.42	0.003943	Down	16.8800	16.1	3	3	5	chloroplast
W9SGP3	Peptide-methionine (R)-S-oxide reductase	L484_011296	0.37	0.001632	Down	15.0490	14.5	2	2	4	chloroplast
W9RAW2	S-adenosylmethionine synthase	L484_025140	0.30	0.000579	Down	43.0380	38.7	11	2	38	cytoskeleton
**12. Biosynthesis of secondary metabolites (29)**											
W9QQ17	Carotenoid cleavage dioxygenase(predicted)	L484_012507	1.53	0.000044	Up	61.1940	7	3	3	3	cytoplasm
W9RXB1	Putative pyridoxal biosynthesis protein PDX1	L484_022003	0.66	0.003788	Down	33.2430	22.9	7	7	25	cytoplasm
W9QU48	Monocopper oxidase-like protein SKU5	L484_019025	0.65	0.000560	Down	66.0630	12.7	5	5	13	plasma membrane
W9SXG6	4-coumarate--CoA ligase-like 7	L484_020607	0.64	0.015450	Down	72.3720	3.6	2	2	3	plasma membrane
W9RHT5	(+)-neomenthol dehydrogenase	L484_012145	0.63	0.034574	Down	61.5880	18.3	7	7	10	chloroplast
W9QK95	ATP phosphoribosyltransferase	L484_018444	0.61	0.003792	Down	43.8100	9.7	3	3	3	chloroplast
W9R3L0	3-isopropylmalate dehydrogenase	L484_025685	0.61	0.004260	Down	33.6340	7.4	3	2	5	mitochondria
W9R7K3	Phenol hydroxylase	L484_007445	0.60	0.000556	Down	18.3650	21.3	4	4	7	chloroplast

	reductase(predicted)									
W9R4J3	Methylenetetrahydrofolate reductase	L484_024666	0.59	0.000032	Down	65.4330	11.5	6	6	8	cytoplasm
W9RAB8	Geranylgeranyl diphosphate reductase	L484_015953	0.59	0.004065	Down	51.8130	11.8	5	5	11	chloroplast
W9SW85	Actin(predicted)	L484_028016	0.58	0.001028	Down	41.6840	44	13	1	85	cytoskeleton
W9RZJ2	Cucumisin-like(predicted)	L484_010382	0.58	0.003030	Down	157.2400	8.1	10	1	47	extracellular
W9R715	3-ketoacyl-CoA synthase	L484_018632	0.57	0.015308	Down	61.1050	3.1	1	1	1	plasma membrane
W9RR73	Polyketide cyclase/dehydrase(predicted)	L484_010725	0.56	0.002260	Down	18.7580	6	1	1	4	chloroplast
W9SYN1	Delta(24)-sterol reductase	L484_014984	0.56	0.001543	Down	65.9730	3.5	2	2	2	cytoplasm
W9RNG3	ATP sulfurylase 2	L484_016033	0.56	0.003618	Down	53.9910	10.2	4	4	5	chloroplast
W9RT19	Deoxyuridine 5'-triphosphate nucleotidohydrolase	L484_021183	0.54	0.001471	Down	18.3200	34.7	4	4	11	cytoplasm
W9SKB4	Brefeldin A-inhibited guanine nucleotide-exchange protein 1	L484_019576	0.51	0.000318	Down	194.1100	0.6	1	1	1	chloroplast
W9SVN5	Actin(predicted)	L484_010751	0.49	0.004345	Down	41.7240	44	13	3	66	cytoskeleton
W9S8U4	Caffeoyl-CoA O-methyltransferase	L484_027504	0.48	0.005798	Down	27.8950	8.1	2	2	3	cytoskeleton
W9RH20	Adenine/guanine permease AZG2	L484_008181	0.47	0.007402	Down	57.6790	1.3	1	1	2	plasma membrane
W9SY03	glycine-rich cell wall structural protein 1(predicted)	L484_012657	0.45	0.003880	Down	13.7560	11.2	1	1	2	chloroplast
W9RLB1	5-methyltetrahydropteroyltriglutamate--homocysteine methyltransferase	L484_003436	0.43	0.000761	Down	83.0980	12	8	4	20	cytoplasm

W9SEL9	Shikimate kinase	L484_005673	0.43	0.002447	Down	29.9320	3.3	1	1	1	chloroplast
W9QYE6	S-adenosylmethionine synthase	L484_013867	0.41	0.005031	Down	43.2240	42	11	3	38	cytoplasm
W9RZ32	Putative 2,3-dimethylmalate lyase(predicted)	L484_018664	0.37	0.001203	Down	31.9450	10.1	2	2	2	cytoplasm
W9R191	Flavoprotein wrbA	L484_026946	0.34	0.010091	Down	27.7700	3.2	1	1	2	cytoplasm
W9QDC3	S-norcoclaurine synthase	L484_019669	0.26	0.003537	Down	17.4210	5.1	1	1	1	cytoplasm
W9R9X3	Ketol-acid reductoisomerase	L484_004803	0.26	0.003037	Down	45.8630	5.9	2	1	4	chloroplast
**13. Others (111)**											
W9RAR9	SHSP domain-containing protein	L484_025169	7.64	0.000059	Up	9.9701	12.8	1	1	6	nucleus
W9T1J8	SHSP domain-containing protein	L484_001270	6.28	0.000039	Up	24.4270	35.9	8	8	26	chloroplast
W9SLI2	DYW_deaminase domain-containing protein	L484_000761	6.00	0.000098	Up	91.3230	5	3	2	18	nucleus
W9RPL6	Uncharacterized protein	L484_026989	5.63	0.000231	Up	8.4156	17.5	1	1	1	chloroplast
W9QE87	SHSP domain-containing protein	L484_006026	4.67	0.000035	Up	17.9940	50.6	9	2	48	cytoplasm
W9S4S5	SHSP domain-containing protein	L484_025214	4.65	0.000030	Up	17.7920	24.1	5	2	16	cytoplasm
W9QWD1	Microtubule-associated protein 1B-like(predicted)	L484_024785	4.56	0.000162	Up	140.1400	0.9	1	1	2	nucleus
W9R6J2	Alpha-amylase/subtilisin inhibitor	L484_010986	3.25	0.022297	Up	22.6520	12.1	2	2	3	extracellular
W9SEX6	VWFA domain-containing protein(predicted)	L484_001964	3.02	0.000643	Up	34.7110	6.5	2	1	3	nucleus
W9SB48	PREDICTED: plant(predicted)	L484_012802	2.99	0.001049	Up	16.8180	5.2	1	1	1	cytoplasm
W9S7S3	Polycystic kidney disease protein 1-like 2	L484_003953	2.96	0.003490	Up	19.4920	12.1	2	2	4	extracellular
W9RTP7	Phytocyanin domain-containing protein	L484_009602	2.90	0.000155	Up	34.5240	12.8	5	5	17	plasma membrane
W9SMH6	Filament-like protein(predicted)	L484_027497	2.88	0.000419	Up	118.9700	1.3	1	1	2	nucleus

W9R5H6	VWFA domain-containing protein	L484_023909	2.87	0.001210	Up	64.9470	7.2	4	3	5	nucleus
W9S839	Alpha-amylase	L484_006340	2.78	0.000161	Up	46.7200	25.2	10	10	26	chloroplast
W9QXX3	C2 domain-containing protein	L484_026371	2.62	0.000092	Up	38.1440	4.1	1	1	2	chloroplast
W9RCI5	SHSP domain-containing protein	L484_027612	2.54	0.000898	Up	25.5470	15.6	4	4	10	chloroplast
W9QR81	SHSP domain-containing protein	L484_016474	2.48	0.000398	Up	22.1930	38.7	6	6	22	chloroplast
W9S016	Alginate_lyase2 domain-containing protein	L484_017854	2.44	0.000082	Up	35.2740	3.5	1	1	1	chloroplast
W9SLC1	Jacalin-type lectin domain-containing protein	L484_016205	2.43	0.000064	Up	15.9570	51.3	6	6	121	cytoplasm
W9RRW7	SHSP domain-containing protein	L484_005024	2.42	0.000221	Up	16.0530	50.4	7	7	20	cytoplasm
W9RKW6	Endochitinase 1	L484_014362	2.40	0.002743	Up	34.8940	33.5	8	1	38	extracellular
W9QZZ7	SHSP domain-containing protein	L484_003777	2.38	0.000130	Up	17.7250	15.7	2	2	39	cytoplasm
W9QPR4	PREDICTED: At5g39570(predicted)	L484_013569	2.31	0.000662	Up	44.6860	21	5	5	22	nucleus
W9SFU7	Uncharacterized protein	L484_006449	2.29	0.000934	Up	8.2167	11.4	1	1	1	chloroplast
W9R1H4	Ricin B-type lectin domain-containing protein	L484_013688	2.27	0.000386	Up	41.3570	5.5	2	2	5	nucleus
W9RE05	Protein FLX-like(predicted)	L484_009687	2.24	0.000511	Up	45.5310	7.1	3	3	4	mitochondria
W9S5T8	PsbP domain-containing protein 4	L484_001712	2.22	0.000258	Up	29.4890	26.2	6	6	25	extracellular
W9RTD9	DAO domain-containing protein	L484_009601	2.19	0.000780	Up	45.0080	5.6	2	2	3	cytoplasm
W9RJ27	PLAT domain-containing protein	L484_003952	2.17	0.000871	Up	20.8980	17.4	3	3	9	vacuolar membrane
W9RD81	PH domain-like(predicted)	L484_010416	2.15	0.000750	Up	23.4250	11.6	2	2	4	cytoplasm
W9RXT2	Uncharacterized protein	L484_024208	2.13	0.001584	Up	9.0470	12.8	1	1	1	nucleus
W9S0V3	AAI domain-containing protein	L484_027077	2.10	0.000112	Up	10.1560	9.2	1	1	10	chloroplast
W9SFV2	ETF_alpha domain-containing	L484_000227	2.09	0.001569	Up	19.0260	23	3	3	5	chloroplast

	protein										
W9S4K7	Kunitz-type serine protease inhibitor	L484_004394	2.08	0.003601	Up	24.6500	4.5	1	1	2	chloroplast
W9QUH8	Putative methyltransferase	L484_005231	2.06	0.002366	Up	30.2970	18.4	6	6	8	cytoplasm
W9RCI6	Putative S-adenosyl-L-methionine-dependent methyltransferase	L484_001600	2.04	0.003331	Up	38.4540	3.5	1	1	2	cytoplasm
W9SDN8	C2H2-type domain-containing protein(predicted)	L484_015202	2.03	0.000599	Up	33.8430	27.7	5	5	19	chloroplast
W9QYW1	DUF953 domain-containing protein	L484_013059	1.97	0.002280	Up	14.9910	45.5	6	6	23	cytoplasm
W9RP37	CS domain-containing protein	L484_006823	1.93	0.000328	Up	35.1830	23.2	8	8	14	nucleus
W9RJ66	Ureohydrolase(predicted)	L484_025588	1.91	0.005989	Up	37.1260	20.9	4	4	5	cytoplasm
W9R6D9	Acid phosphatase 1	L484_026293	1.87	0.010446	Up	29.6220	6.5	2	2	4	chloroplast
W9SLU4	Inhibitor I9 domain-containing protein	L484_000622	1.86	0.001259	Up	11.1840	27.4	3	1	15	nucleus
W9S1E1	Inhibitor I9 domain-containing protein	L484_003889	1.85	0.006471	Up	11.0140	29.2	3	1	13	chloroplast
W9RH73	Ornithine aminotransferase	L484_005329	1.83	0.003715	Up	26.9350	13.6	3	3	6	chloroplast
W9RVC1	L-idonate 5-dehydrogenase	L484_005351	1.82	0.000403	Up	39.5520	10.6	4	4	11	cytoplasm
W9R048	PREDICTED: fiber Fb15(predicted)	L484_016868	1.76	0.002788	Up	10.5120	27	3	3	7	cytoplasm
W9SJA0	Protein FLX-like(predicted)	L484_004540	1.76	0.000000	Up	31.7260	26.1	7	7	14	mitochondria
W9R6P7	NifU-like protein 1	L484_018769	1.74	0.006132	Up	23.5980	5.5	1	1	1	chloroplast
W9S1G2	Embryo-specific(predicted)	L484_012965	1.68	0.017024	Up	20.7820	20.8	3	3	12	chloroplast
W9SCL4	Rossmann-like alpha/beta/alpha sandwich fold containing	L484_026631	1.68	0.007338	Up	23.3090	28.3	5	5	13	chloroplast

	protein(predicted)										
W9S5P2	Sin3 associated polypeptide p(predicted)	L484_020767	1.65	0.006396	Up	15.9610	33.1	5	2	16	cytoplasm
W9RX20	ANK_REP_REGION domain-containing protein	L484_022581	1.65	0.004749	Up	22.0740	6.3	1	1	3	cytoplasm
W9R5Y6	Selenium-binding protein 2	L484_026870	1.64	0.000673	Up	72.6130	13.1	8	2	26	cytoplasm
W9QQR1	WEB family(predicted)	L484_023722	1.62	0.001294	Up	87.0820	4.7	3	3	3	chloroplast
W9SIE4	LRRNT_2 domain-containing protein	L484_028068	1.61	0.000428	Up	37.3010	31	10	9	36	extracellular
W9QWH6	DUF3456 domain-containing protein(predicted)	L484_024837	1.60	0.015098	Up	23.8190	23.3	5	5	12	extracellular
W9RRE2	Furry(predicted)	L484_019074	1.59	0.007910	Up	14.8460	16.2	2	2	5	chloroplast
W9QP43	RING-type domain-containing protein	L484_006018	1.58	0.000059	Up	143.8800	2	2	2	4	nucleus
W9R005	Uncharacterized protein	L484_000610	1.58	0.000038	Up	7.8920	21.9	1	1	7	cytoplasm
W9RWG0	Aldo_ket_red domain-containing protein	L484_021750	1.56	0.000211	Up	40.2970	26.3	10	10	36	cytoplasm
W9QG21	PREDICTED: CISIN_1g031376mg(predicted)	L484_016939	1.53	0.003217	Up	18.6960	5.8	1	1	2	nucleus
W9RQ50	DUF538 domain-containing protein(predicted)	L484_016699	1.51	0.000637	Up	18.7800	15	2	2	7	chloroplast
W9SMK9	EKC/KEOPS complex subunit Tprkb-like(predicted)	L484_027533	0.66	0.007637	Down	18.8210	5.8	1	1	1	nucleus
W9QN08	Peptidase_S9 domain-containing protein(predicted)	L484_010276	0.66	0.000976	Down	23.2480	5.1	1	1	2	chloroplast
W9RLT9	FabA domain-containing protein	L484_024122	0.66	0.005989	Down	25.6720	9.4	2	1	3	chloroplast

W9RDH0	GRAS domain-containing protein	L484_003031	0.66	0.011251	Down	66.6820	1.6	1	1	1	chloroplast
W9R5B3	MHD domain-containing protein	L484_012850	0.65	0.008236	Down	69.3780	2.6	2	2	3	cytoplasm
W9QFK5	FAS1 domain containing protein(predicted)	L484_018219	0.65	0.006234	Down	44.0780	14.6	4	4	19	chloroplast
W9SZM7	CUGBP Elav-like family member 5	L484_020442	0.65	0.023028	Down	47.3260	4.6	2	2	3	nucleus
W9RZD6	AAI domain-containing protein	L484_010375	0.64	0.001009	Down	12.2110	8.6	1	1	2	extracellular
W9RA90	Protein SET	L484_015928	0.64	0.000372	Down	22.4760	17.5	4	4	6	cytoplasm
W9SEN2	HMA domain-containing protein	L484_003497	0.64	0.006258	Down	25.3750	5.1	1	1	9	chloroplast
W9RR24	ZmSiR protein	L484_015306	0.62	0.000201	Down	77.3340	8.1	5	5	5	cytoplasm
W9R3Z3	AB hydrolase-1 domain-containing protein	L484_025738	0.61	0.023991	Down	43.5530	16.1	4	4	6	chloroplast
W9R3X0	Zinc finger protein MAGPIE	L484_022919	0.61	0.001200	Down	57.5610	5.9	2	2	2	nucleus
W9RAA6	PREDICTED: CICLE_v10032810mg(predicted)	L484_004245	0.61	0.004317	Down	20.3080	10.2	1	1	1	chloroplast
W9RK79	Malectin_like domain-containing protein(predicted)	L484_018192	0.60	0.004370	Down	12.1390	8.3	1	1	2	extracellular
W9S4Y5	F9L1.21 protein(predicted)	L484_000574	0.59	0.004952	Down	6.9450	32.8	2	2	5	nucleus
W9SSW6	Extended synaptotagmin-3	L484_021894	0.59	0.000205	Down	61.0710	6	4	4	4	cytoplasm
W9RVE9	TCTP domain-containing protein	L484_023512	0.59	0.000209	Down	18.8420	56	6	6	14	cytoplasm
W9R3A0	PSII_BNR domain-containing protein	L484_018094	0.58	0.001274	Down	45.3100	30.4	11	11	22	chloroplast
W9RW23	SAM domain-containing protein	L484_007030	0.58	0.006079	Down	28.3350	6.3	1	1	2	nucleus
W9RYZ1	AP-2 complex subunit mu	L484_023279	0.58	0.001232	Down	67.4650	1.7	1	1	1	plasma membrane
W9SBY5	Endoplasmin-like protein	L484_021880	0.58	0.000173	Down	114.0100	22.6	22	22	40	chloroplast
W9SET6	ANK_REP_REGION	L484_000390	0.57	0.007743	Down	19.5410	8.9	1	1	6	chloroplast

	domain-containing protein										
W9RA88	PKS_AT domain-containing protein	L484_011151	0.57	0.000057	Down	42.7440	9.8	4	4	6	chloroplast
W9S356	Pentatricopeptide repeat(predicted)	L484_020794	0.54	0.004742	Down	45.6790	3.5	1	1	1	cytoplasm
W9T005	Uncharacterized protein	L484_020052	0.54	0.000544	Down	19.8840	14.9	3	3	7	nucleus
W9QX35	LRR domain containing protein(predicted)	L484_008883	0.54	0.010417	Down	48.8900	3.9	2	2	4	chloroplast
W9RWC1	WPP domain-associated protein(predicted)	L484_026005	0.52	0.002822	Down	104.4900	1	1	1	1	nucleus
W9S383	PREDICTED: CICLE_v10028680mg(predicted)	L484_008432	0.51	0.002227	Down	46.8530	12	5	5	7	chloroplast
W9R548	Aa_trans domain-containing protein	L484_002148	0.49	0.047501	Down	45.4720	1.7	1	1	3	cytoplasm
W9SDM9	LRRNT_2 domain-containing protein	L484_000482	0.48	0.002191	Down	40.1440	14.3	4	4	5	cytoplasm
W9R393	Tubulin beta chain	L484_024283	0.48	0.003990	Down	50.0780	10.6	4	1	19	nucleus
W9S956	Protein HOTHEAD	L484_023132	0.47	0.000987	Down	63.0120	7.1	4	4	4	extracellular
W9R8S5	Uncharacterized protein	L484_008689	0.46	0.004586	Down	9.5998	21.7	1	1	4	chloroplast
W9RQZ1	ADP,ATP carrier protein	L484_015275	0.45	0.001703	Down	41.9970	18.3	7	3	25	chloroplast
W9SZ70	Porphobilinogen deaminase	L484_007545	0.45	0.000424	Down	40.9230	22.6	8	8	21	chloroplast
W9QP38	AAI domain-containing protein	L484_020879	0.45	0.003443	Down	14.3620	13	2	2	2	vacuolar membrane
W9SBN1	Chromodomain-helicase-DNA-binding protein 5	L484_018514	0.43	0.009449	Down	256.7300	0.4	1	1	1	nucleus
W9QZW3	Tubulin beta chain	L484_006042	0.43	0.002535	Down	50.4740	20.5	7	1	23	nucleus

W9RD59	MLP-like protein 423	L484_027819	0.41	0.000934	Down	17.5750	57.7	9	9	57	extracellular
W9SRT8	Sperm-associated antigen 1	L484_005244	0.40	0.001529	Down	49.2760	2.6	1	1	1	nucleus
W9S7F7	Tubulin beta chain	L484_006496	0.40	0.000213	Down	50.3210	28.2	10	1	31	nucleus
W9QTJ4	FabA domain-containing protein	L484_016604	0.40	0.003031	Down	23.7100	14.8	3	2	5	cytoplasm
W9RWX0	TPM_phosphatase domain-containing protein(predicted)	L484_024338	0.35	0.001026	Down	10.5850	34	2	2	4	chloroplast
W9RHA4	Armadillo-type fold containing protein(predicted)	L484_011017	0.34	0.002002	Down	71.0960	1.4	1	1	3	chloroplast
W9S4V7	P-loop containing nucleoside triphosphate hydrolase(predicted)	L484_019460	0.33	0.001465	Down	48.9660	8.5	3	3	4	chloroplast
W9RCN4	Cell division topological specificity factor-like protein	L484_004236	0.32	0.015167	Down	25.9070	3	1	1	2	chloroplast
W9QDK1	LRAT domain-containing protein	L484_019724	0.29	0.000284	Down	10.4130	14.1	1	1	1	chloroplast

[*]The fold change (drought/control) over 1.5 was considered up-regulation; whereas the fold change less than 1/1.5 (0.67) was considered down-regulation (Student's *t*-test, $P < 0.05$).
