# Supplementary material for: Quantitative Proteomics and Functional Characterization Reveal That Glutathione Peroxidases Act as Important Antioxidant Regulators in Mulberry Response to Drought Stress
Source: Plants (Basel). 2022 Sep 8;11(18):2350. doi: 10.3390/plants11182350 (PMC9500794; doi:10.3390/plants11182350)
Supplement: Supplementary file 1 [file plants-11-02350-s001.zip › Table S3.pdf]

Table S1. Primer sequences used for qRT-PCR in this study.

Primer name	Primer sequences (5′ to 3′)
MaActin-F	GAGCAAGGAGATCACAGCCC
MaActin-R	CCAGACTCGTCGTACTCGC
AtActin-F	GGTAACATTGTGCTCAGTGGTGG
AtActin-R	AACGACCTTAATCTTCATGCTGC
MaGPX1-F	CTGAAGTCAAGCAAGGGAGG
MaGPX1-R	CTAAGAGGAGAAGTGGTGGGAG
MaGPX2-F	GTTGAGGTGAATGGGAAGGATG
MaGPX2-R	TTGAGAGGAGATGTGGTAGGAGC
MaGPX3-F	GGGGACAACATCAAGTGGAAC
MaGPX3-R	AAGAGGGGAAGTGGTAGGTGC
MaGPX4-F	GCAGGAGGTTTCTTAGGTGATGT
MaGPX4-R	AAGGAGATGTTGTTGGTGGGT
MaGPX5-F	GCACGGGCACTGCTCCTT
MaGPX5-R	CACGCTTCTCCCAATGCTTTC
MaGPX6-F	CGTTATTACCCAACAACTTCTG
MaGPX6-R	GCTTCTTTATGTCACGCTCAA
