# Supplementary figures and images for: Quantitative Proteomics and Functional Characterization Reveal That Glutathione Peroxidases Act as Important Antioxidant Regulators in Mulberry Response to Drought Stress
Source: Plants (Basel). 2022 Sep 8;11(18):2350. doi: 10.3390/plants11182350 (PMC9500794; doi:10.3390/plants11182350)

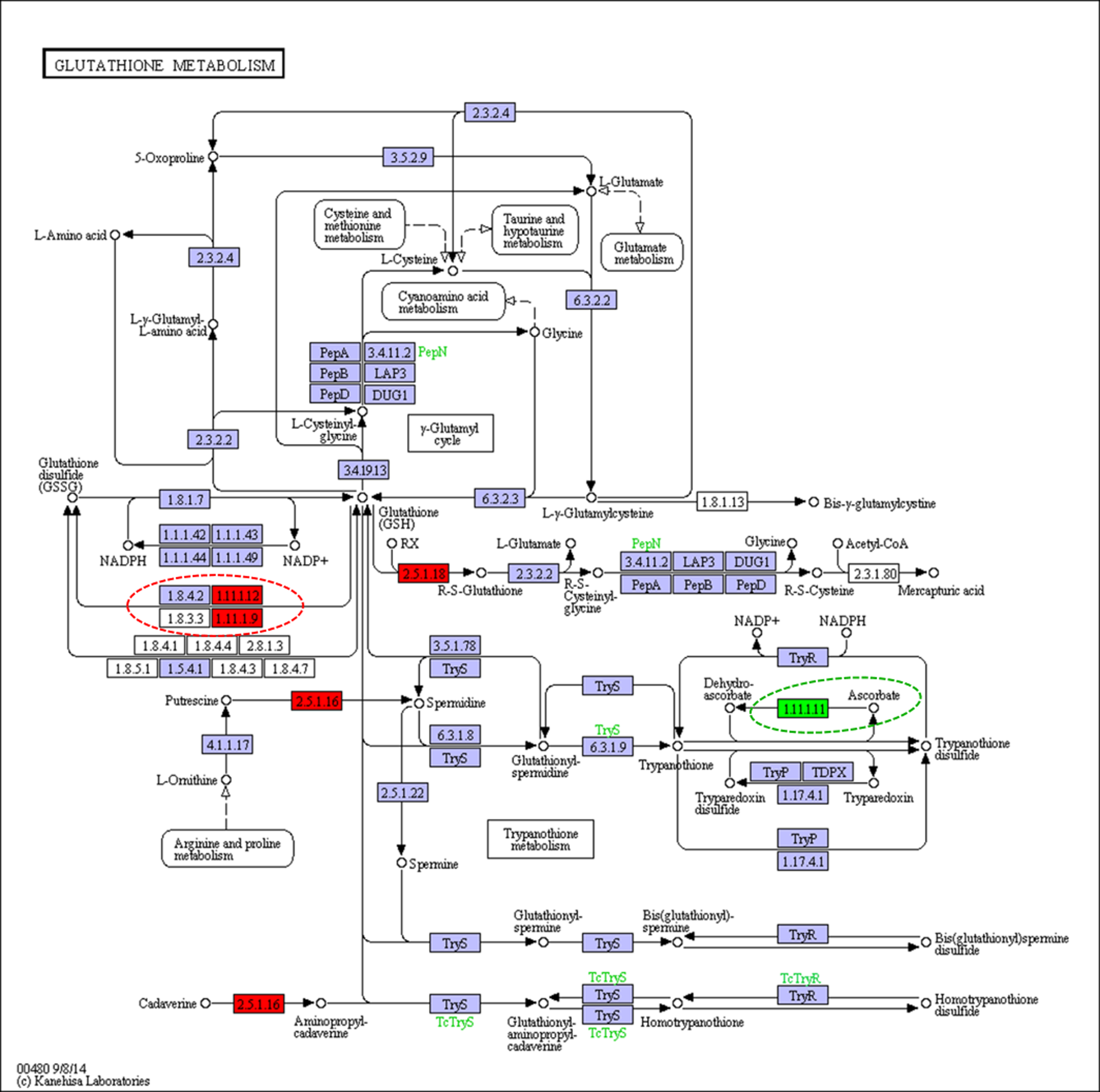

Supplement: Supplementary file 1 [file plants-11-02350-s001.zip › Figure S1.tif]

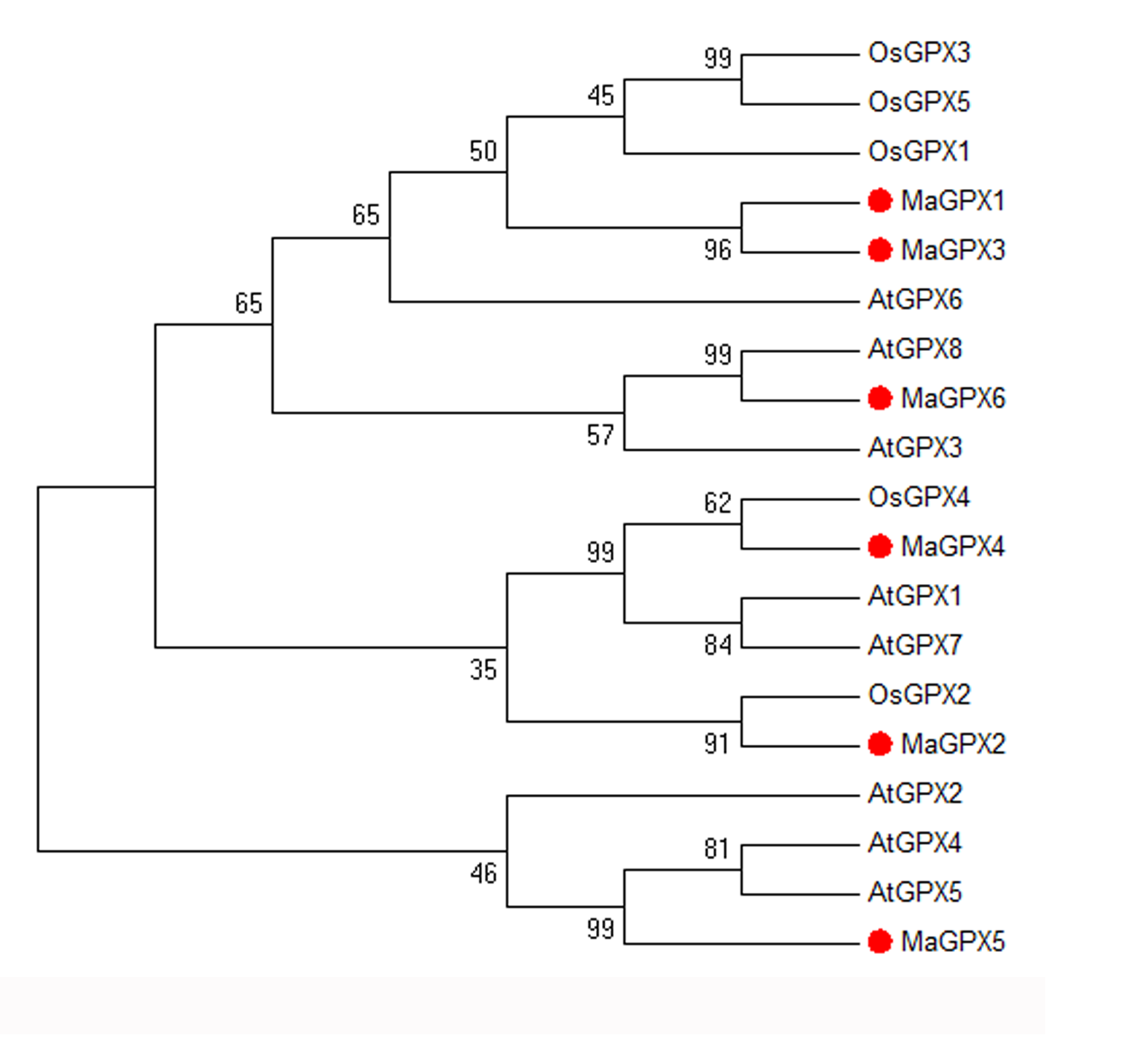

Supplement: Supplementary file 1 [file plants-11-02350-s001.zip › Figure S2.tif]

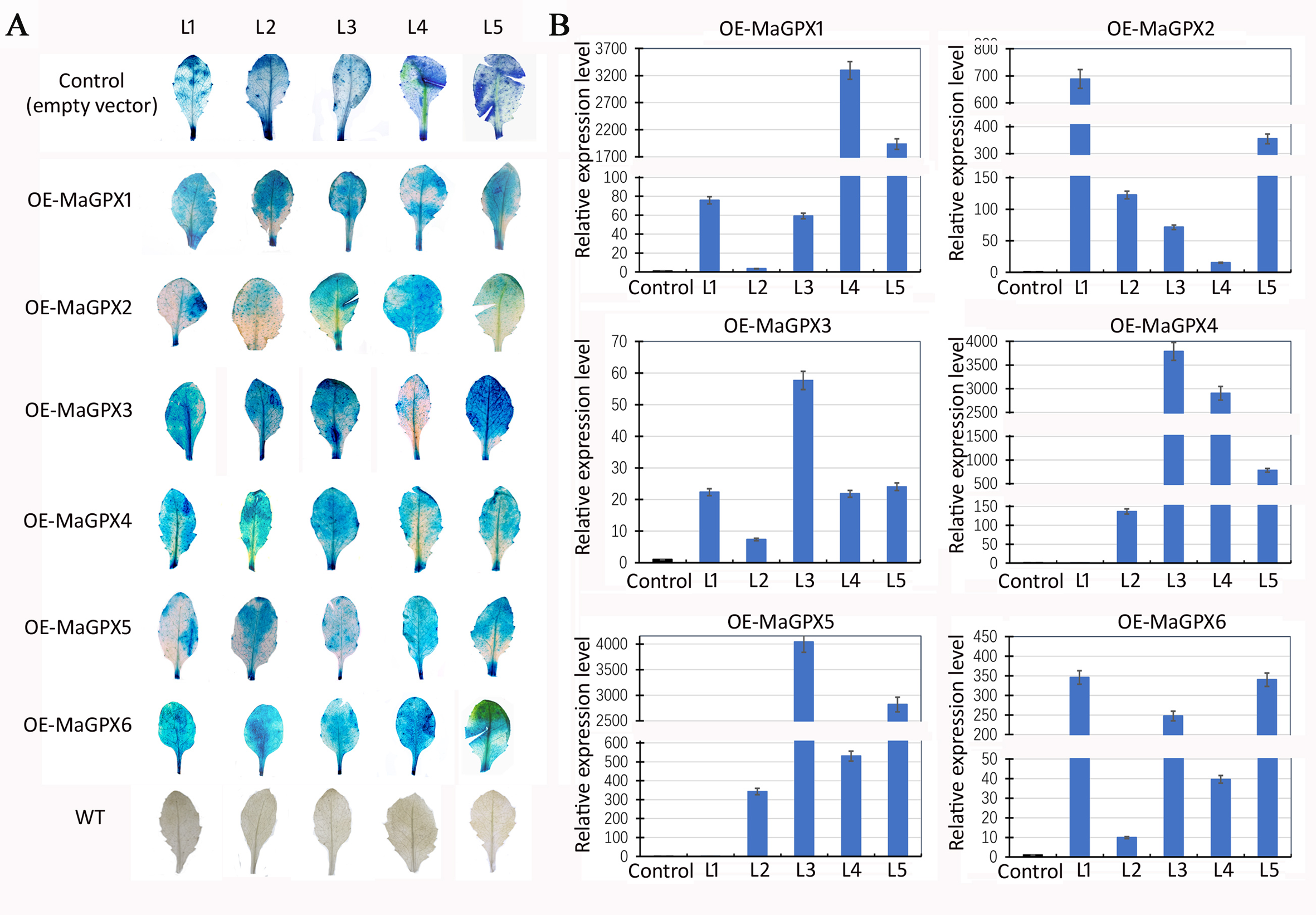

Supplement: Supplementary file 1 [file plants-11-02350-s001.zip › Figure S3.tif]

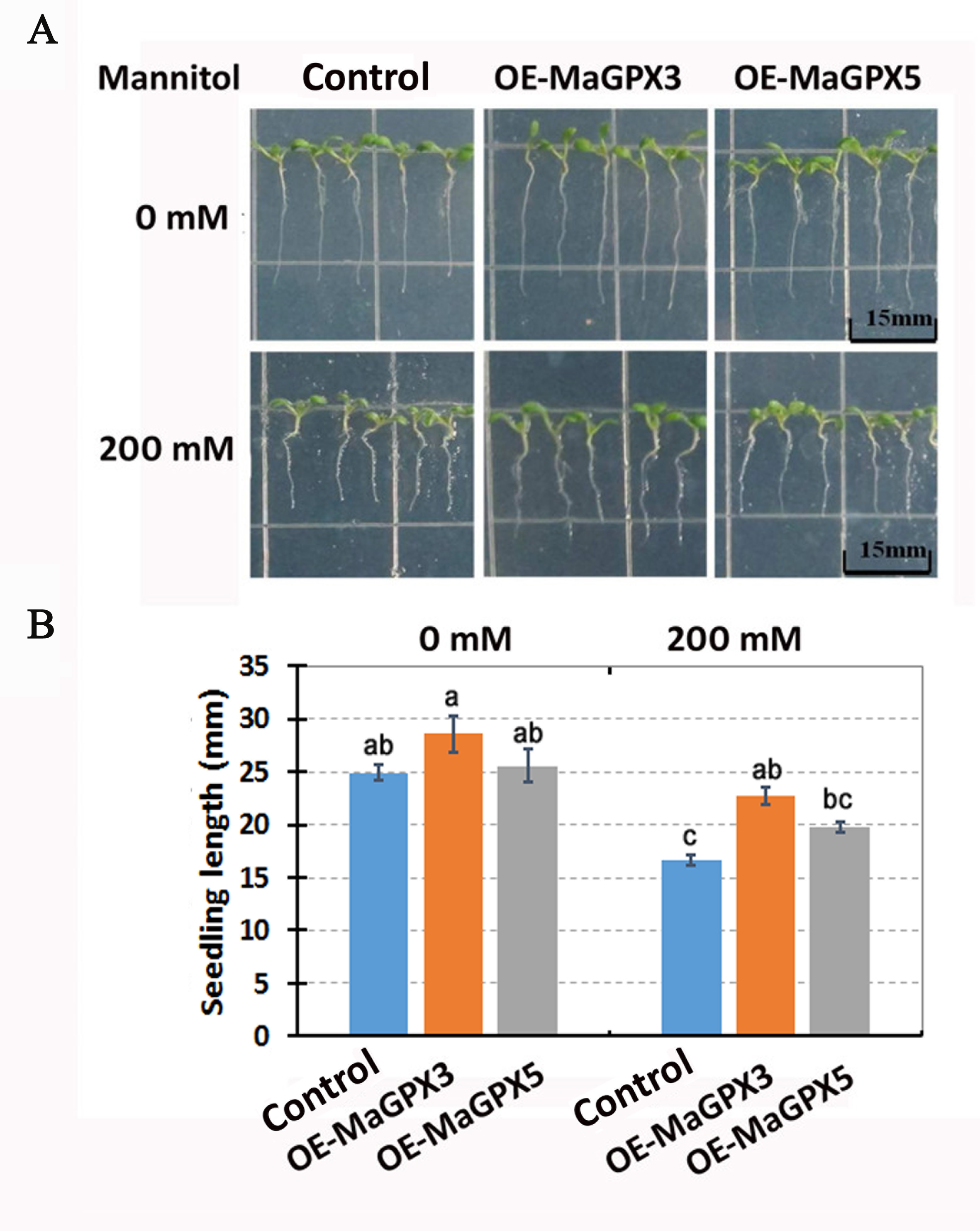

Supplement: Supplementary file 1 [file plants-11-02350-s001.zip › Figure S4.tif]
